# Network pharmacology mechanisms and experimental verification of *Hedyotis Diffusae Herba-Scutellariae Barbatae Herba* drug pair extract in the treatment of nasopharyngeal carcinoma

**DOI:** 10.3389/fonc.2025.1601725

**Published:** 2025-06-18

**Authors:** Tiantian Li, Haijun Chen, Jing Zhang, Jiaxin Liu, Rundong Tang, Liang Zhang, Tian Feng, Yanchun Xiao, Shiwen Liu, Xiangjun Chen

**Affiliations:** ^1^ Department of Otorhinolaryngology, Shenzhen Bao’an Traditional Chinese Medicine Hospital Group, Guangzhou University of Chinese Medicine, Shenzhen, China; ^2^ School of Pharmaceutical Sciences, Hunan University of Medicine, Huaihua, China; ^3^ Department of Oncology, Shenzhen Bao’an Traditional Chinese Medicine Hospital Group, Guangzhou University of Chinese Medicine, Shenzhen, China; ^4^ Department of Acupuncture, Shenzhen Bao’an Traditional Chinese Medicine Hospital Group, Guangzhou University of Chinese Medicine, Shenzhen, China; ^5^ Department of Dermatology, Shenzhen Bao’an Traditional Chinese Medicine Hospital Group, Guangzhou University of Chinese Medicine, Shenzhen, China

**Keywords:** drug pair, Hedyotis Diffusae Herba, nasopharyngeal carcinoma, network pharmacology, PI3K/Akt signaling pathway, Scutellariae barbatae herba

## Abstract

**Background:**

Nasopharyngeal carcinoma (NPC) represents the predominant head-neck malignancy in China. While the Hedyotis Diffusae Herba-Scutellariae Barbatae Herba (HDH-SBH) herb pair shows antitumor potential, its mechanism against NPC remains unclear.

**Methods:**

This network pharmacology study integrated with experimental validation identified NPC-related targets through GEO database and disease databases (OMIM, GeneCards, TTD). Active components of HDH-SBH and their targets were retrieved from Traditional Chinese Medicine Systems Pharmacology Database (TCMSP). Common targets were analyzed via STRING, with functional enrichment using Gene Ontology (GO) and Kyoto Encyclopedia of Genes and Genomes (KEGG). Core components were validated through molecular docking and *in vitro* experiments using HDH-SBH-treated 5-8F and CNE2 cells.

**Results:**

We identified 36 bioactive components and 155 shared targets, with quercetin, luteolin, wogonin, and β-sitosterol emerging as core components. KEGG analysis highlighted PI3K/AKT pathway inhibition (P<0.05). Molecular docking confirmed strong binding between core components and key targets (AKT1, TP53, BCL2). *In vitro* validation showed HDH-SBH significantly inhibited NPC cell proliferation/migration while inducing apoptosis through downregulating BCL2, upregulating TP53, and suppressing AKT1 phosphorylation.

**Conclusions:**

Based on the network pharmacology approach, we predicted the potential mechanism of HDH-SBH for the treatment of NPC, which provided a new idea for further research on its pharmacological mechanism.

## 1 Introduction

Nasopharyngeal carcinoma (NPC), a malignancy of the head and neck, originates from the epithelial cells lining the nasopharyngeal mucosa. It predominantly manifests in the pharyngeal recesses and the anterior superior wall of the nasopharynx, potentially presenting with a range of clinical manifestations such as retrograde epistaxis, nasal congestion, tinnitus, auditory deficits, cephalalgia, among other symptoms, distinguished by its insidious progression (1). The People’s Republic of China stands as a predominant epicenter for NPC, harboring approximately 80% of the global incidence. Data procured from the International Agency for Research on Cancer, a subsidiary of the World Health Organization, indicated a burden of 62,000 new diagnoses and 34,000 mortalities attributable to NPC within China in the annum 2020 (2). Present therapeutic paradigms for NPC predominantly entail a synergistic approach involving radiotherapy coupled with chemotherapy. Nevertheless, recurrence and distal metastasis emerge as pivotal deterrents to successful treatment outcomes (3–6). Noteworthy adverse effects associated with chemoradiotherapy encompass salivary gland impairment, radiodermatitis, and otic and nasal damage (7). The quest for therapeutic modalities surpassing the extant standards, thereby amplifying the safety and efficaciousness of NPC treatment, constitutes an exigent clinical quandary.

**Figure 1:**
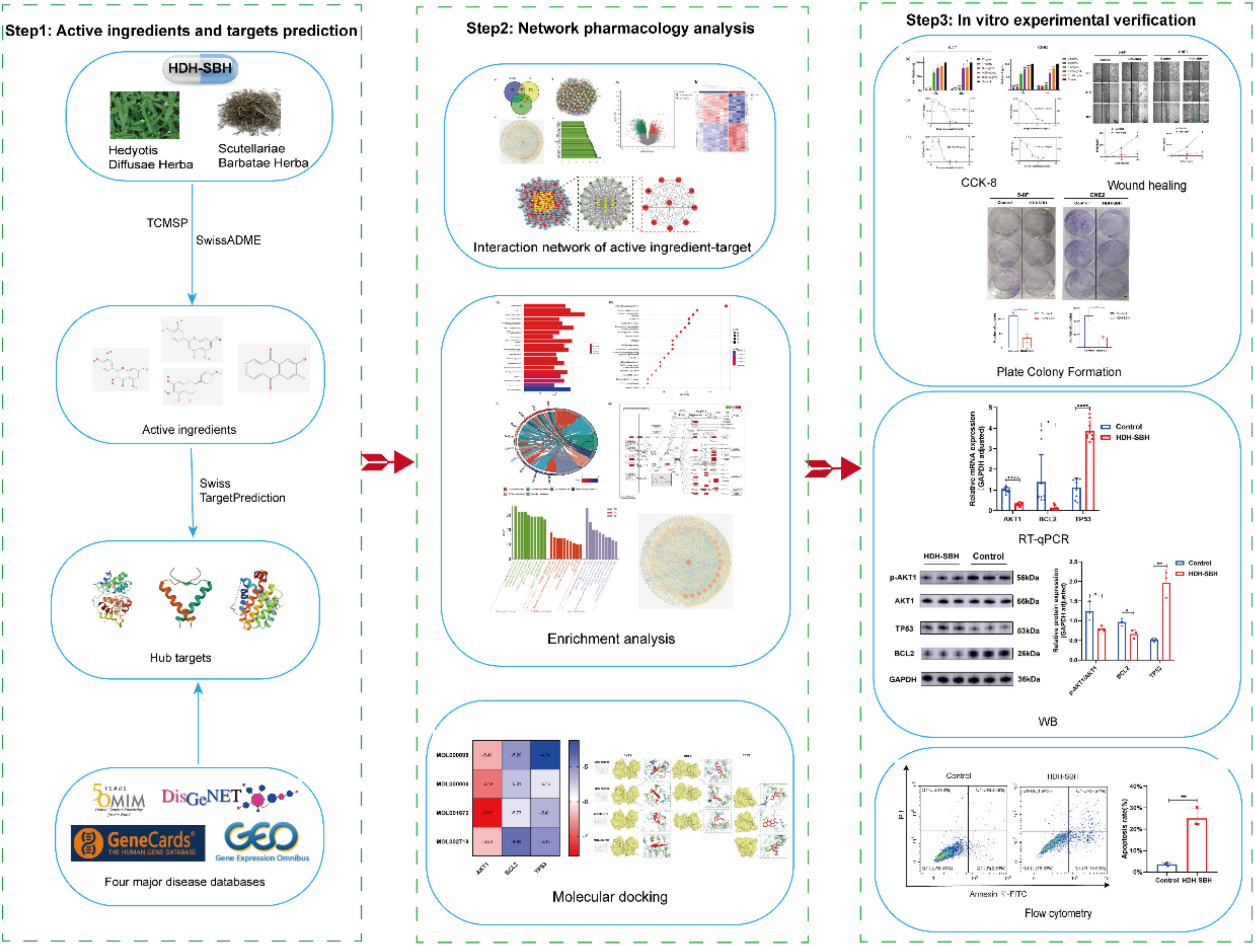
The process of this research. The OMIM icon was obtained from https://omim.org/; the DisGeNET icon from https://disgenet.com/; the GeneCards icon from https://www.genecards.org/; and the GEO icon from https://www.ncbi.nlm.nih.gov/geo/.

The exploration of antineoplastic agents within the realm of Traditional Chinese Medicine (TCM) is receiving burgeoning interest, attributed to their potential in tumor suppression alongside a diminished profile of toxic side effects and a lower propensity for resistance development vis-à-vis conventional chemotherapy agents (8). Oncological formulations in TCM frequently incorporate dyadic herbal combinations, underpinned by a wealth of clinical evidence, theoretical constructs, and specific formulation tenets (9, 10). Amongst these, the *Hedyotis Diffusae Herba-Scutellariae Barbatae Herba* (HDH-SBH) duo stands as a quintessential anticancer drug pair in clinical praxis. HDH, sourced from the desiccated entirety of Oldenlandia diffusa (Willd.) Roxb., is characterized by a mildly bitter and sweet gustatory profile, cool temperament, acclaimed for its properties in clearing heat, detoxification, resolving dampness, and diuresis. Contemporary pharmacological inquiries have substantiated HDH’s prowess in attenuating tumor cell proliferation, catalyzing apoptosis, impeding angiogenesis, modulating immune responses, alongside its anti-inflammatory, antioxidative, and autophagy-inducing capabilities (11–13). SBH, procured from the desiccated entirety of Lobelia chinensis Lour., is noted for its pungent flavor and neutral nature, and lauded for its heat-clearing, detoxifying, dampness-resolving, and anti-edematous properties. Recent pharmacological investigations have illuminated SBH’s effectiveness in fostering tumor cell apoptosis, mitigating cancer cell glycolysis, and attenuating cancer cell drug resistance (14–16). However, scholarly examinations concerning the operative mechanisms of HDH-SBH drug pair in the prophylaxis and treatment of NPC remain sparse. Consequently, this study seeks to delineate the molecular underpinnings of HDH-SBH drug pair’s efficacy against NPC through an amalgamation of GEO microarray data, network pharmacology, and molecular docking methodologies, corroborated by *in vitro* cellular assays, to elucidate the potential role of HDH-SBH drug pair duo in the prevention and management of NPC, thereby establishing a foundational corpus for ensuing scholarly endeavors (**Figure 1**).

## 2 Materials and methods

### 2.1 Acquisition of GEO differential targets

Leveraging “Nasopharyngeal carcinoma” as a focal keyword, a methodical search was initiated within the Gene Expression Omnibus (GEO) repository (https://www.ncbi.nlm.nih.gov/geo/). The inclusion criteria were specified as “Homo” for the species and “Expression profiling by array” for the method of expression analysis. This resulted in the acquisition and subsequent analysis of high-throughput sequencing datasets, employing R 4.2.1 software for the meticulous process of analysis and filtration, adopting absolute values of log fold change (|logFC|) greater than 1 and adjusted P-values (P_adj_) less than 0.05 as stringent parameters for discerning differential gene expression. This analytical rigor culminated in the creation of both volcano plots and heatmaps, visual representations that succinctly convey the significant disparities in gene expression.

### 2.2 Acquisition of NPC targets

In a parallel vein, the keyword “Nasopharyngeal carcinoma” served as the linchpin for identifying therapeutic targets pertinent to NPC across three eminent disease databases: GeneCards (https://www.genecards.org/), Disgenet (https://www.disgenet.org/search), and the Online Mendelian Inheritance in Man (OMIM) database (https://omim.org/search/advanced/geneMap). A diligent process ensued to eliminate duplicate gene entries, with the resulting disease target proteins undergoing standardization through the UniProt database (https://www.uniprot.org), ensuring uniformity and precision in target identification.

### 2.3 Screening of active ingredients and targets for HDH-SBH drug pair

Via the Traditional Chinese Medicine Systems Pharmacology Database and Analysis Platform (TCMSP, https://old.tcmsp-e.com/tcmsp.php), active ingredients of HDH-SBH drug pair were identified using “Hedyotis Diffusae Herba” and “Scutellariae Barbatae Herba” as keywords. Active ingredients were selected based on pharmacokinetic properties, including absorption, distribution, metabolism, and excretion (ADME), with oral bioavailability (OB) ≥30% and drug-likeness (DL) ≥0.18 as criteria. Potential therapeutic targets of these active ingredients were obtained through the TCMSP and SwissTargetPrediction platforms (http://www.swisstargetprediction.ch/) and standardized via the UniProt database.

### 2.4 Selection of common targets and construction of the PPI network

To synthesize the therapeutic landscape, the Venny 2.1.0 platform was employed to intersect the sets of potential therapeutic targets of HDH-SBH drug pair, differentially expressed genes from the GEO analysis, and NPC disease targets, thus identifying commonalities and generating Venny diagrams. These intersecting targets were then introduced into the STRING database (https://cn.string-db.org/), choosing “Homo sapiens” as the species and adopting a medium confidence threshold with a combined score greater than 0.4, for the construction of a Protein-Protein Interaction (PPI) network. This network was subsequently imported into the Cytoscape 3.7.2 software to perform network characteristic analysis, where variations in the size and color of the nodes visually encoded the Degree values, elucidating a hierarchical structure of target significance within the network. Nodes of larger size and darker hue denoted higher Degree values, marking them as pivotal in the network’s topology. The apex of this analytical journey was reached with the identification of the top 10 targets through the MCODE plugin in Cytoscape 3.7.2 software, delineating the core targets implicated in HDH-SBH drug pair’s therapeutic efficacy against NPC.

### 2.5 Construction of the “drug-ingredient-target-disease” interaction network

Nodes representing drugs, active ingredients, diseases, and common targets were defined and their relationships established in an Excel spreadsheet. This data was then imported into Cytoscape 3.7.2 software to create an interaction network diagram. The Network Analyzer plugin was utilized for topological analysis, calculating the degree, betweenness centrality (BC), and closeness centrality (CC) of each node. Core active ingredients were identified by setting thresholds at the median values of BC and CC, and then ranking them according to their degree values.

### 2.6 GO functional analysis and KEGG enrichment analysis

The R 4.2.1 software was employed to retrieve the entrez IDs of the common targets. GO analysis, focusing on the function of gene products, and KEGG Pathway enrichment analysis, emphasizing the extent of core pathway enrichment, were conducted on these targets (17). The common targets were uploaded into the R 4.2.1 software, with parameters set as pvalueCutoff = 0.05 and qvalueCutoff = 0.05. After completing the enrichment analysis, the clustering network was exported, and results were ranked based on P-values. The Top 10 GO enrichment results and Top 20 KEGG Pathway enrichment results were visualized as corresponding bubble plots and bar charts using ImageGP (http://www.bic.ac.cn/BIC/#/) (18, 19). These analyses aimed to further explore the potential biological functions and signaling pathway mechanisms of HDH-SBH drug pair in combating NPC.

### 2.7 Molecular docking validation

Molecular docking was performed between the core active ingredients (small molecule ligands) and core targets (large molecule receptors). The molecular structures of these core active ingredients and core targets were downloaded from the TCMSP and PDB databases (https://www.rcsb.org/) respectively, and saved in mol2 and pdb formats. These structures were imported into AutoDock Tools 1.5.6 software for processing. After exporting the files in pdbqt format, docking was conducted to validate the interactions.

### 2.8 *In vitro* experimental validation

#### 2.8.1 Cell line and experimental drugs

The 5-8F nasopharyngeal carcinoma cell line was obtained from Zhejiang Meisen Cell Technology Co., Ltd. (Zhejiang, China); the CNE2 cell line was acquired from Qingqi Biotechnology Development Co., Ltd. (Shanghai, China). All cell lines underwent STR authentication (BNCC341932, BNCC341794). All cell lines were cultured in RPMI-1640 medium enriched with 10% FBS and 1% penicillin-streptomycin. Subsequently, the cells were placed in an incubator adjusted to a tem-perature of 37^◦^C, harboring a humid atmosphere comprising 5% CO_2_. The traditional Chinese medicines HDH, sourced from Ji’an, Jiangxi, with the batch number A211216, and SBH, obtained from Fuyang, Anhui, with the batch number A220728, were investigated.

#### 2.8.2 Key reagents and instruments

Fetal bovine serum (FBS) was purchased from GIBCO, and the Cell Counting Kit-8 (CCK-8) was acquired from Biosharp (batch number BS350B). Western Blot reagents were obtained from Beyotime Biotech Inc. The study utilized several instruments, including a DSZ2000X fluorescence microscope (Zhongxian Hengye Co., Ltd., Beijing), a DH-160ICO2 incubator (Santeng Instrument Equipment Co., Ltd., Shanghai), an EIX808U enzyme-linked immunosorbent assay (ELISA) reader (BioTek, USA), and a CytoFLEX flow cytometer (BECKMAN COULTER, USA). Additional equipment included a DYCZ-24DH electrophoresis apparatus, a DYY-7C membrane transfer apparatus (Liuyi Biotechnology Co., Ltd., Beijing), and a ChemiScope 6100 chemiluminescence imaging system (Qinxian Scientific Instruments Co., Ltd., Shanghai).

#### 2.8.3 Extraction and preparation of HDH-SBH drug pair

50 g of HDH and 50 g of SBH were added to a 2 L distillation flask, followed by the addition of 800 mL of 70% ethanol. The flask was then placed in a constant temperature water bath at 95°C for the first extraction for 1 hour. After the extraction, the liquid was decanted, and another 600 mL of 70% ethanol was added for a second extraction in the constant temperature water bath for 0.5 hours. The extracts from both extractions were combined and concentrated using a rotary evaporator at 60°C and a speed of 60 rpm until solid precipitate formed. The extract was then pre-cooled in a -20°C freezer before being transferred to a lyophilizer for further concentration to dry powder. The yield of the lyophilized powder was 9.45 g, representing a yield of 9.45% (calculated at 100% concentration). HDH-SBH drug pair lyophilized powder was dissolved in ultrapure water to prepare a solution with a concentration of 8 mg/mL, and it was filtered through a 0.22 µm pore size filter to remove bacteria for subsequent use.

#### 2.8.4 CCK-8 assay

Cells in the logarithmic growth phase, 5-8F and CNE2, were adjusted to a cell density of 5,000 cells/well in 100 µL of complete culture medium and seeded into a 96-well plate. The plate was incubated at 37°C in an atmosphere of 5% CO_2_ with saturated humidity for 24 hours. HDH-SBH drug pair concentration gradient was set at 2 mg/mL, 1 mg/mL, 0.5 mg/mL, 0.25 mg/mL, 0.125 mg/mL, and 0 mg/mL, added to the aforementioned 96-well plate with 12 replicate wells per group, along with a blank control group without drugs or cells. The plate was further incubated for 24 and 48 hours, with medium changes every 24 hours. Subsequently, the complete culture medium was mixed with the CCK-8 solution in a 9:1 ratio, added to each well avoiding light, and incubated for 2 hours in a constant temperature incubator. Finally, the 96-well plate was placed in an ELISA reader to measure the absorbance at 450 nm, and the cell proliferation rate was calculated. The cell proliferation rate = (Absorbance of drug-treated wells - Absorbance of blank wells)/(Absorbance of control wells - Absorbance of blank wells) * 100%. The IC_50_ of the drug was calculated using GraphPad Prism 8.0 based on the drug concentration and cell proliferation rate.

#### 2.8.5 Plate colony formation assay

A density of 500 cells/well of NPC cells was seeded into a 6-well plate and cultured for 24 hours to allow cell adhesion. Three replicates were set for both the experimental and control groups; the experimental group was treated with the IC_50_ concentration of the drug determined from the 48-hour treatment, while the control group received complete culture medium. After 48 hours, the medium was replaced with complete culture medium containing 10% FBS and continued until visible clone colonies were observed, at which point the culture was terminated. The cells were fixed with 4% paraformaldehyde for 15 minutes and stained with 0.1% crystal violet solution for 15 minutes, then gently washed with water and air-dried at room temperature. The colony formation rate was calculated as (Number of colonies/Number of seeded cells) × 100%.

#### 2.8.6 Wound healing assay

Parallel lines were evenly drawn on the back of the plate with a marker pen, with an average distance of 5 mm between adjacent lines. Log-phase NPC cells were adjusted to a density of 50,000 cells/mL and seeded at 2 mL/well in a 6-well plate. After 24 hours of culture, the experimental group was treated with the IC_50_ concentration of the drug from the 48-hour treatment, while the control group received complete culture medium. A 200 μL pipette tip was used to scratch vertically across the cell monolayer, followed by washing three times with PBS and addition of serum-free medium. Images were taken at 0, 24, and 48 hours under an inverted microscope, and the wound closure distance was analyzed using ImageJ 1.5.3 software. The cell migration rate was calculated as: Migration rate (%) = (Width of the initial scratch - Width of the scratch at the respective time point)/Width of the initial scratch × 100%.

#### 2.8.7 qRT-PCR

Log-phase NPC cells were adjusted to a density of 50,000 cells/mL and seeded in 2 mL of complete culture medium in a 6-well plate, and incubated at 37°C in a 5% CO_2_ incubator for 24 hours. Three replicates were set for both the experimental and control groups; the experimental group received the IC_50_ concentration of the drug from the 48-hour treatment, while the control group received complete culture medium. After co-culturing for 48 hours, total RNA was extracted using the Trizol method, and the purity of the extracted RNA was determined by a nucleic acid spectrophotometer, with an A260/A280 ratio of 1.8-2.0 indicating high purity. Reverse transcription was performed according to the kit instructions, with primer sequences as shown in **Table 1**. GAPDH was used as an internal reference, and data were recorded and saved following the reaction.

**Table 1:** The primer sequences of qRT-PCR.

Gene	Forward primer(s) (5’ to 3’)	Reverse primer(s) (5’ to 3’)
AKT1	CAAGGTGATCCTGGTGAA	CGTGGGTCTGGAAAGAGT
BCL2	CTTCGCCGAGATGTCCAGC	CCCAGTTCACCCCGTCCCT
TP53	GCTGGGGCTCCTTCTTGGT	ACCAAGAAGGAGCCCCAGC
GAPDH	CTGGGCTACACTGAGCACC	AAGTGGTCGTTGAGGGCAATG

#### 2.8.8 Western blot

The experimental groups and interventions were consistent with those described in Section 2.8.7. After 48 hours of co-culture, 200 μL of cell lysis buffer was added to each well. The cells were incubated on ice for 5 minutes, then scraped and transferred to 1.5 mL EP tubes. The samples were centrifuged at 12,000 rpm for 15 minutes at 4°C to collect the supernatant for total protein extraction. Protein concentrations were quantified using the BCA assay. The proteins were separated by gel electrophoresis, transferred to membranes, and blocked. Subsequently, the membranes were incubated with primary antibodies (AKT1 [R23412, 1:1000], p-AKT1 [R22961, 1:1000], TP53 [10442-1-AP, 1:5000], BCL2 [12789-1-AP, 1:5000]) and a secondary antibody (511203, 1:10,000). After development, the bands were analyzed using ImageJ software. The relative protein expression levels were calculated as the ratio of the target protein gray value to the internal reference (e.g., GAPDH) gray value.

#### 2.8.9 Apoptosis

Grouping and intervention measures were identical to *qRT-PCR* experiments. After 48 hours of co-culture, cells were digested with trypsin without EDTA and washed twice with pre-cooled PBS. Cells were centrifuged at 4°C for 5 minutes, resuspended in 500 µL of 1× Binding Buffer, and stained with 5 µL Annexin V-FITC and 5 µL PI Staining Solution. The mixture was gently mixed and incubated in the dark at room temperature for 10 minutes before flow cytometry analysis.

### 2.9 Data analysis

All data were processed and analyzed using SPSS 26.0 statistical software, with continuous variables expressed as the mean ± standard deviation (SD). Graphical representations were generated using GraphPad Prism 8.0. Prior to statistical analysis, all datasets underwent normality testing (Shapiro-Wilk test) and homogeneity of variance verification. For comparisons between two groups, independent samples t-tests were applied when data met normality and variance homogeneity assumptions; otherwise, the Mann-Whitney U test (non-parametric) was utilized. In multi-group comparisons, one-way ANOVA was performed for normally distributed data with homogeneous variance, followed by Ryan-Holm step-down Bonferroni *post hoc* tests if significant overall differences were detected. For non-normally distributed or heteroscedastic data, the Kruskal-Wallis H test (non-parametric) was employed, with subsequent Dunn’s multiple comparison tests for pairwise analysis. All statistical tests were two-tailed, and a *P <*0.05 was considered statistically significant. Each experimental group included ≥3 biological replicates, and all experiments were independently repeated three times to ensure robustness and reproducibility.

## 3 Results

### 3.1 Bioinformatics analysis results

#### 3.1.1 Acquisition of active ingredients and candidate targets of HDH-SBH drug pair

Utilizing the TCMSP database, supplemented by literature search and retrospective verification, a total of 7 active ingredients of HDH and 29 active ingredients of SBH were identified, as detailed in **Table 2**. Through predictions and selections in the TCMSP and SwissTargetPrediction databases, followed by standardization in the UniProt database and removal of duplicate targets, a total of 152 potential targets for the active ingredients of HDH and 223 potential targets for the active ingredients of SBH were obtained.

**Table 2:** The main active ingredients of HDH-SBH.

Herb	Ingredient number	Name	OB%	DL
HDH	MOL001646	2,3-dimethoxy-6-methyanthraquinone	34.86	0.26
HDH	MOL001659	Poriferasterol	43.83	0.76
HDH	MOL001663	oleanolic acid	32.03	0.76
HDH	MOL001670	2-methoxy-3-methyl-9,10-anthraquinone	37.83	0.21
HDH	MOL000449	Stigmasterol	43.83	0.76
HDH	MOL000358	beta-sitosterol	36.91	0.75
HDH	MOL000098	quercetin	46.43	0.28
SBH	MOL001040	(2R)-5,7-dihydroxy-2-(4-hydroxyphenyl)chroman-4-one	42.36	0.21
SBH	MOL012245	5,7,4’-trihydroxy-6-methoxyflavanone	36.63	0.27
SBH	MOL012246	5,7,4’-trihydroxy-8-methoxyflavanone	74.24	0.26
SBH	MOL012248	5-hydroxy-7,8-dimethoxy-2-(4-methoxyphenyl)chromone	65.82	0.33
SBH	MOL012250	7-hydroxy-5,8-dimethoxy-2-phenyl-chromone	43.72	0.25
SBH	MOL012251	Chrysin-5-methylether	37.27	0.2
SBH	MOL012252	9,19-cyclolanost-24-en-3-ol	38.69	0.78
SBH	MOL002776	Baicalin	40.12	0.75
SBH	MOL012254	campesterol	37.58	0.71
SBH	MOL000953	CLR	37.87	0.68
SBH	MOL000358	beta-sitosterol	36.91	0.75
SBH	MOL012266	rivularin	37.94	0.37
SBH	MOL001973	Sitosteryl acetate	40.39	0.85
SBH	MOL012269	Stigmasta-5,22-dien-3-ol-acetate	46.44	0.86
SBH	MOL012270	Stigmastan-3,5,22-triene	45.03	0.71
SBH	MOL000449	Stigmasterol	43.83	0.76
SBH	MOL000173	wogonin	30.68	0.23
SBH	MOL001735	Dinatin	30.97	0.27
SBH	MOL001755	24-Ethylcholest-4-en-3-one	36.08	0.76
SBH	MOL002714	baicalein	33.52	0.21
SBH	MOL002719	6-Hydroxynaringenin	33.23	0.24
SBH	MOL002915	Salvigenin	49.07	0.33
SBH	MOL000351	Rhamnazin	47.14	0.34
SBH	MOL000359	sitosterol	36.91	0.75
SBH	MOL005190	eriodictyol	71.79	0.24
SBH	MOL005869	daucostero_qt	36.91	0.75
SBH	MOL000006	luteolin	36.16	0.25
SBH	MOL008206	Moslosooflavone	44.09	0.25
SBH	MOL000098	quercetin	46.43	0.28

#### 3.1.2 Acquisition of GEO differential genes

**Figure 2:**
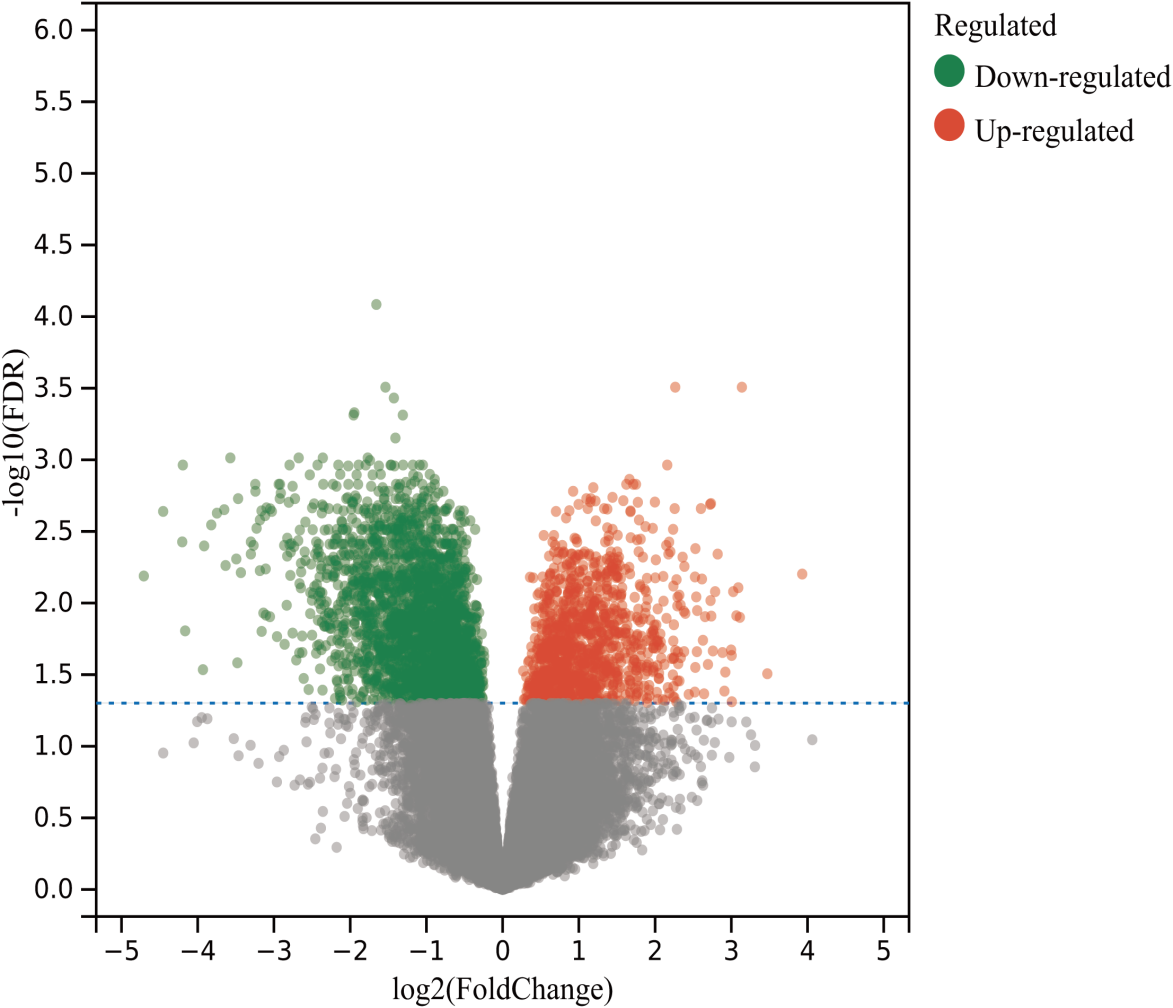
Volcano plots of differentially expressed genes in the GSE118719 dataset of NPC.

**Figure 3:**
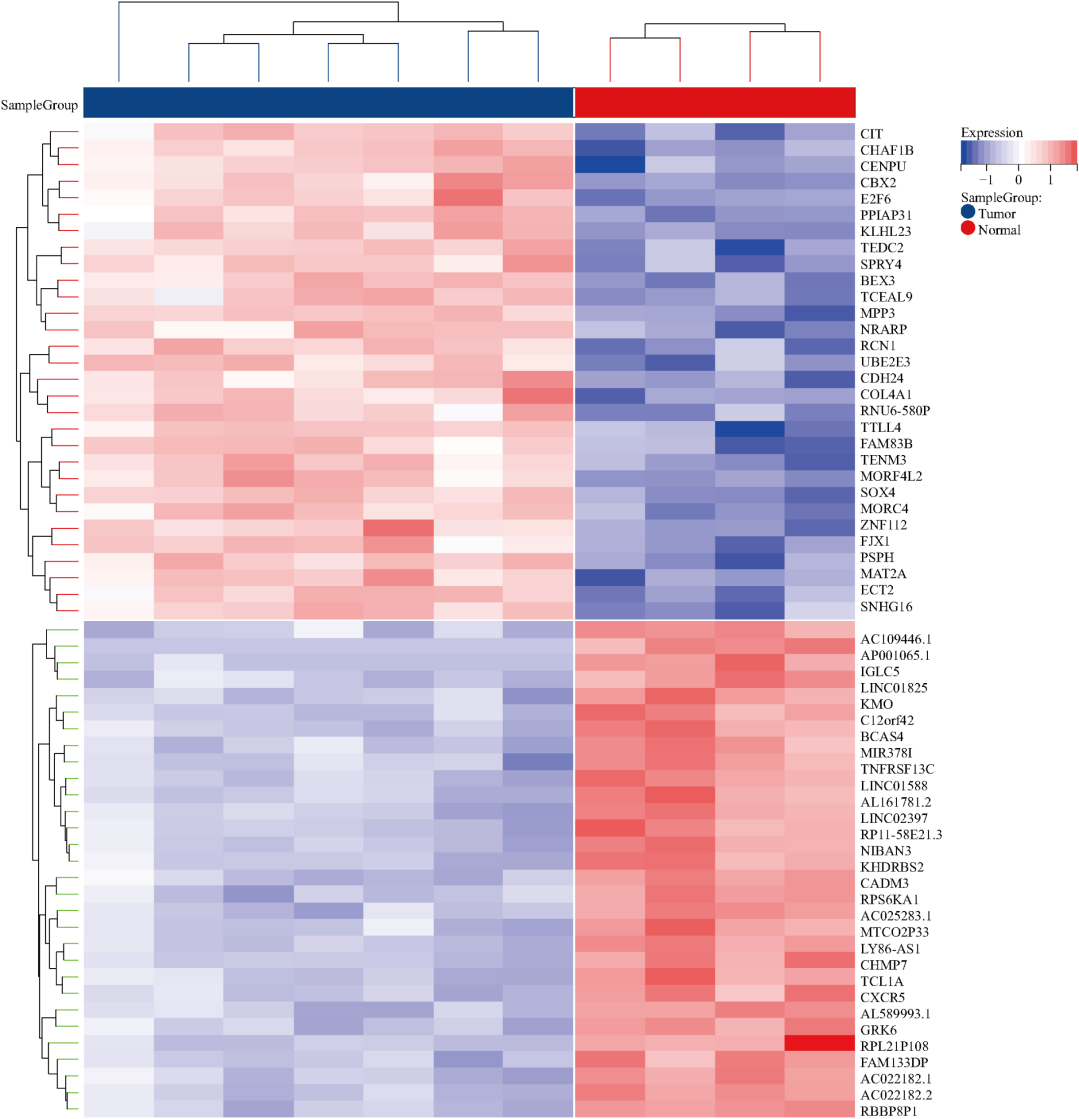
Heatmaps of differentially expressed genes in the GSE118719 dataset of NPC.

By searching the GEO database, the dataset GSE118719 was identified. This microarray dataset encompasses gene expression profiles from 4 normal nasopharyngeal tissues and 7 NPC tissues. Using R software to download and analyze the raw data, 240 differentially expressed genes in NPC were identified based on the criteria of *P*_adj_<0.05 and |logFC|>1. This included 157 downregulated genes and 83 upregulated genes. Volcano plots were created using R, as shown in **Figure 2**. Heatmaps of the top 20 upregulated and 20 downregulated genes are depicted in **Figure 3**.

#### 3.1.3 Identification of potential therapeutic targets for NPC

**Figure 4:**
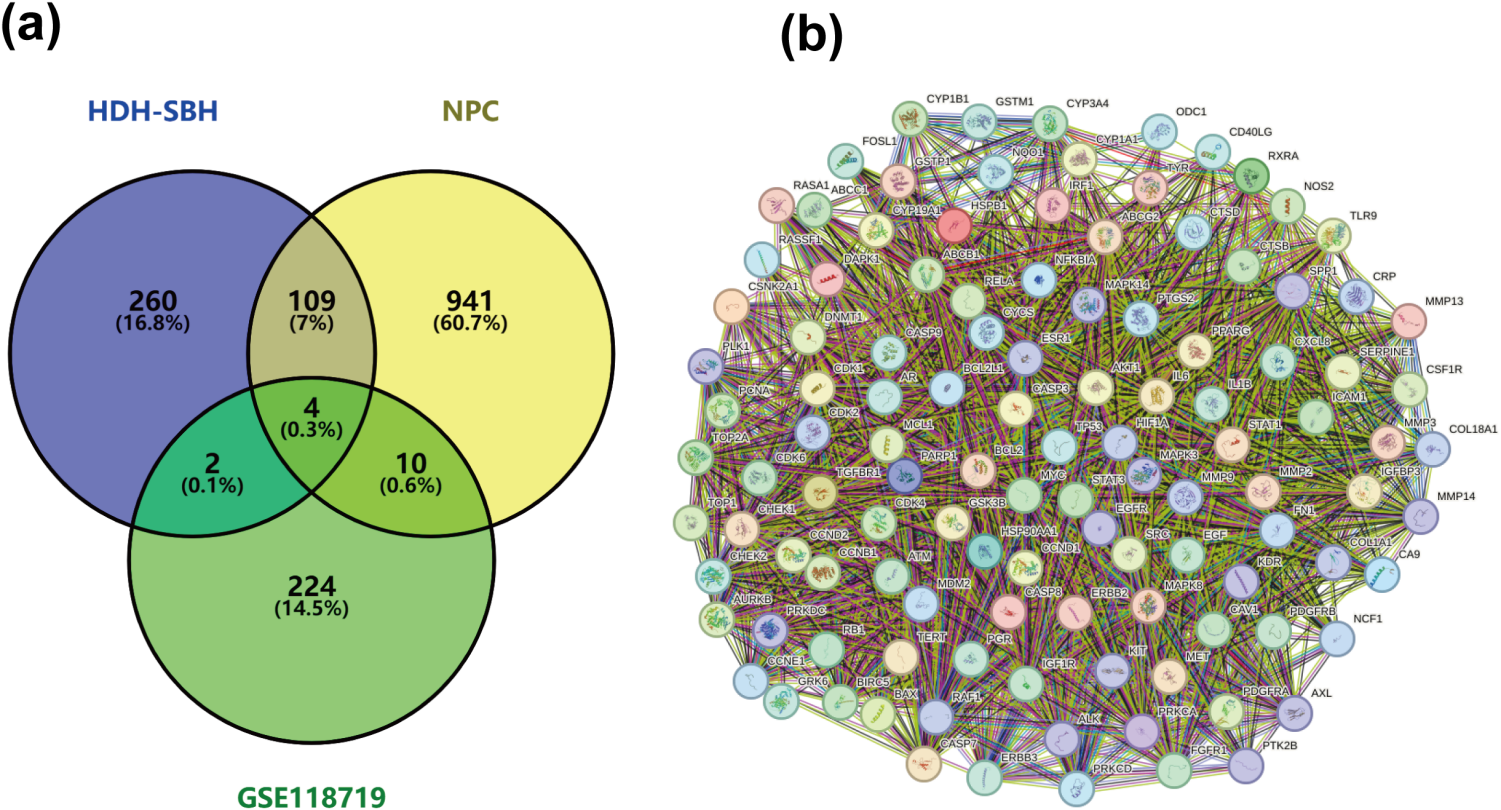
**(a)** Common targets of HDH-SBH drug pair in treating NPC; **(b)** Interaction diagram of HDH-SBH drug pair with common target proteins in NPC.

**Figure 5:**
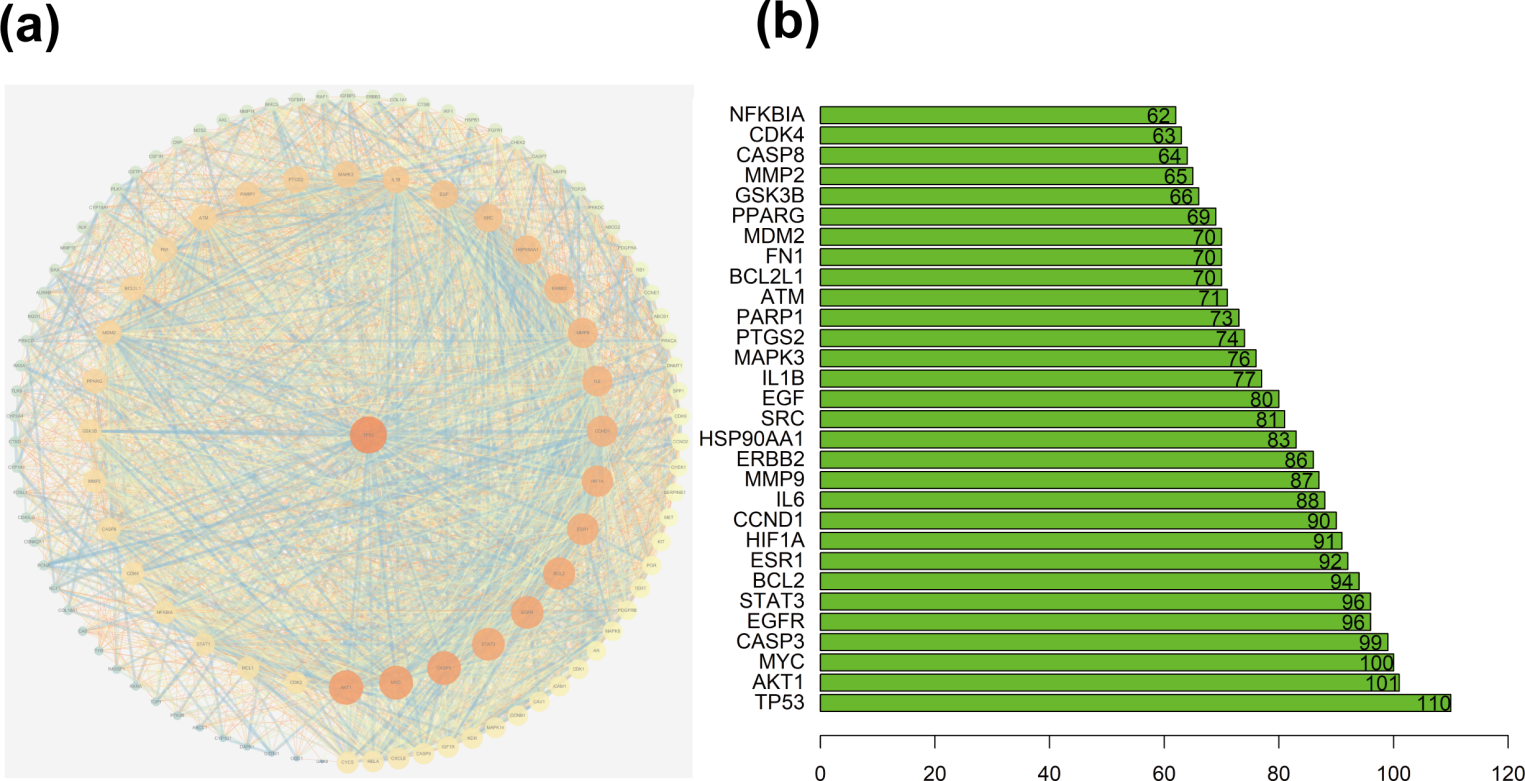
**(a)** Protein interaction network centered on TP53; **(b)** Ranking chart of connectivity frequency of common targets.

**Figure 6:**
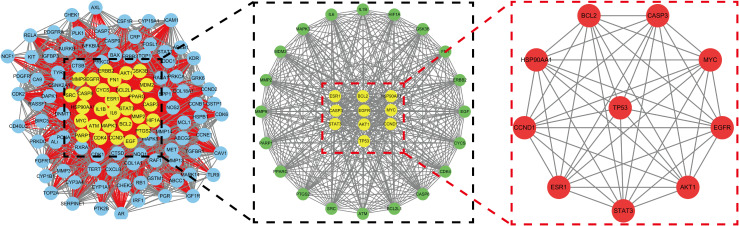
Mesh diagram of the top ten targeted screening. Nodes represent targets, lines represent interactions between targets, the more lines, the more interactions between targets.

A total of 1064 potential therapeutic targets for NPC were obtained from three major disease databases: GeneCards, Disgenet, and OMIM. Common targets between HDH-SBH drug pair and NPC potential targets were identified using the Venny database, yielding 115 common targets, as shown in **Figure 4a**. These 115 common targets were inputted into the STRING database to generate a network diagram of the relationships between these target proteins. This network diagram was visualized using Cytoscape software, as shown in **Figure 4b**. Target network associations were centered around the top-ranked target TP53, as illustrated in **Figure 5a**. The count.R plugin in R software was used to calculate the connection frequencies of the common targets, and the top 30 protein targets were visualized, as shown in **Figure 5b**. The top 10 core proteins identified were TP53, AKT1, MYC, CASP3, EGFR, STAT3, BCL2, ESR1, HIF1A, and CCND1. Network analysis was further conducted using the CytoNCA plugin in Cytoscape, focusing on betweenness centrality (BC), closeness centrality (CC), and degree centrality (DC) as indicators for target selection, as depicted in **Figure 6**.

#### 3.1.4 Construction of drug-ingredient-target-disease network

**Figure 7:**
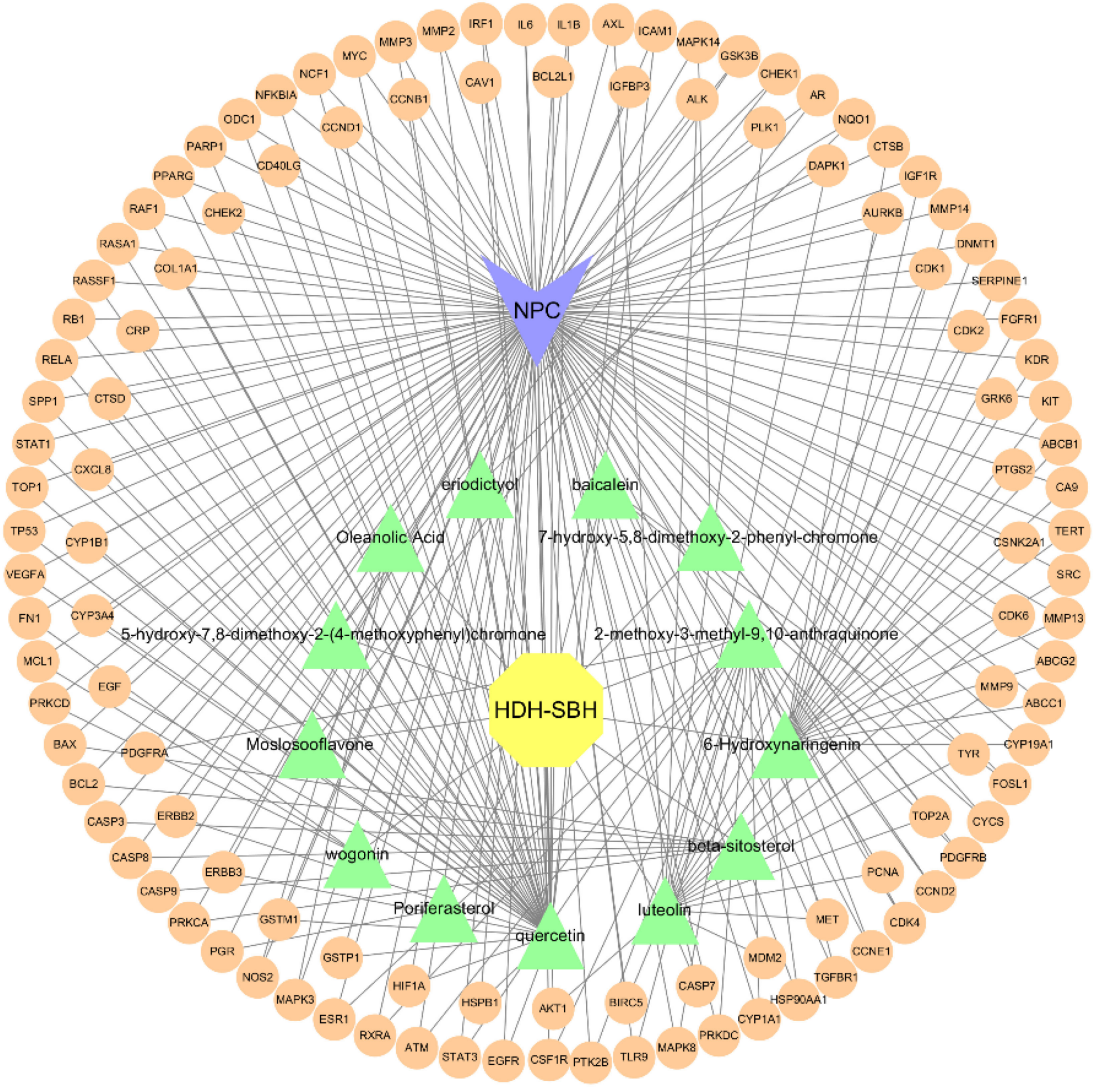
“Drug-Ingredient-Target-Disease” network diagram for HDH-SBH drug pair in the treatment of NPC.

The potential mechanism of action of HDH-SBH drug pair in treating NPC was visualized using Cytoscape software, constructing a network diagram of active ingredients, common targets, and the disease (**Figure 7**). Which includes 130 nodes and 243 edges. In the diagram, orange circles represent shared targets, yellow hexagons represent HDH-SBH drug pair, green triangles represent active ingredients, and purple V-shapes represent NPC. The denser the connections, the more significant the node is within the network. The active ingredients are ranked by the number of edges, from highest to lowest, as follows: quercetin, luteolin, 2-methoxy-3-methyl-9,10-anthraquinone, and 6-Hydroxynaringenin.

#### 3.1.5 GO enrichment analysis and KEGG pathway enrichment analysis

**Figure 8:**
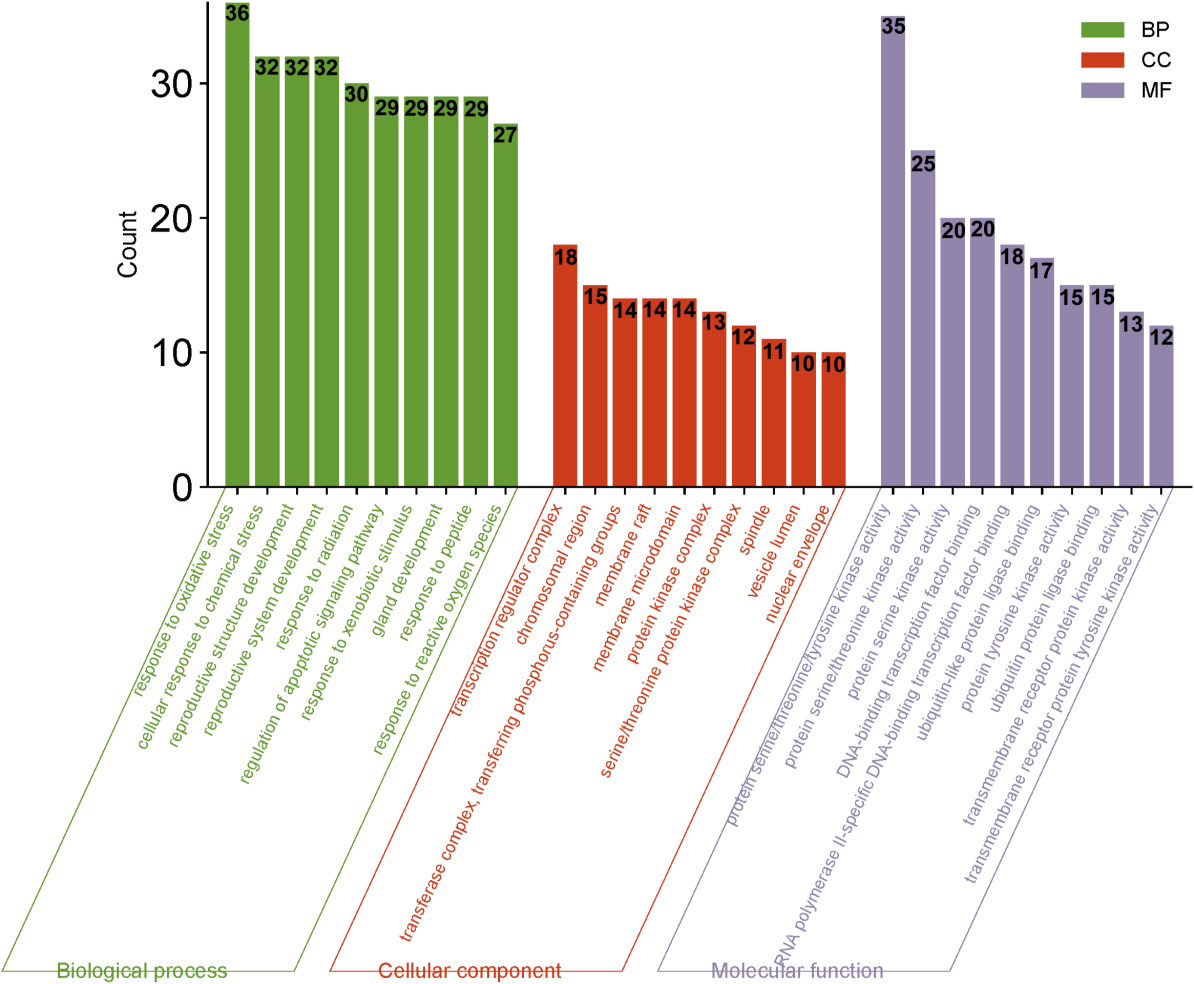
Bar chart of GO analysis for HDH-SBH drug pair in the treatment of NPC.

GO and KEGG analyses were conducted using the Bioconductor bioinformatics package in R software. A total of 2182 GO terms were obtained. Within the biological process (BP) category, 1991 entries primarily related to response to oxidative stress, cellular response to chemical stress, response to reactive oxygen species, and response to radiation, indicating that HDH-SBH drug pair can exert anti-cancer effects through regulating tumor cell responses to various stressors and biological processes. For cellular components (CC), 57 entries were identified, mainly including protein kinase complex, serine/threonine protein kinase complex, and cyclin-dependent protein kinase holoenzyme complex. In the molecular function (MF) category, 134 entries were mainly associated with protein serine/threonine/tyrosine kinase activity, transmembrane receptor protein kinase activity, and DNA-binding transcription factor binding. The top 10 entries in BP, CC, and MF categories were selected for visualization, as shown in **Figure 8**.

**Figure 9:**
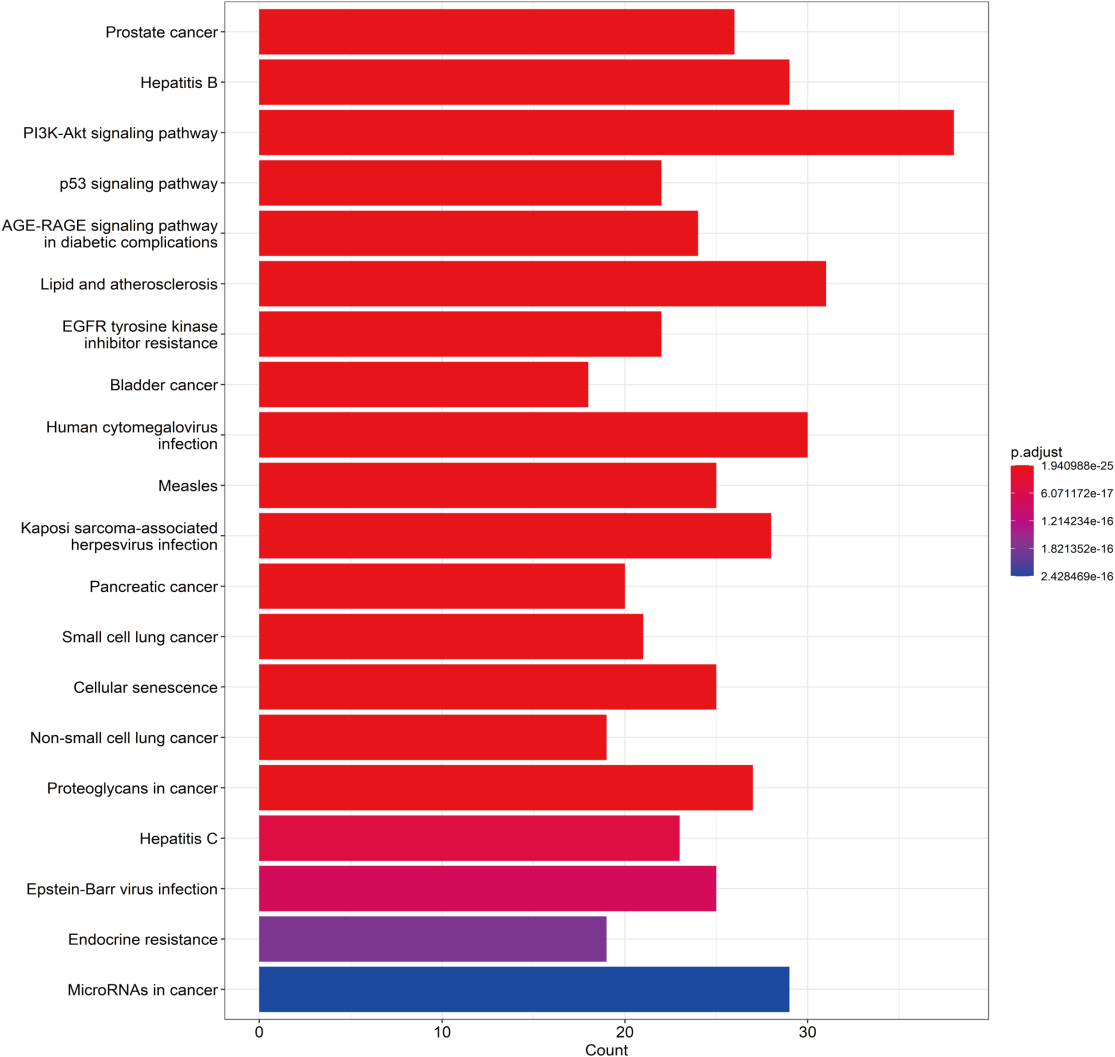
Top 20 KEGG bar charts.

**Figure 10:**
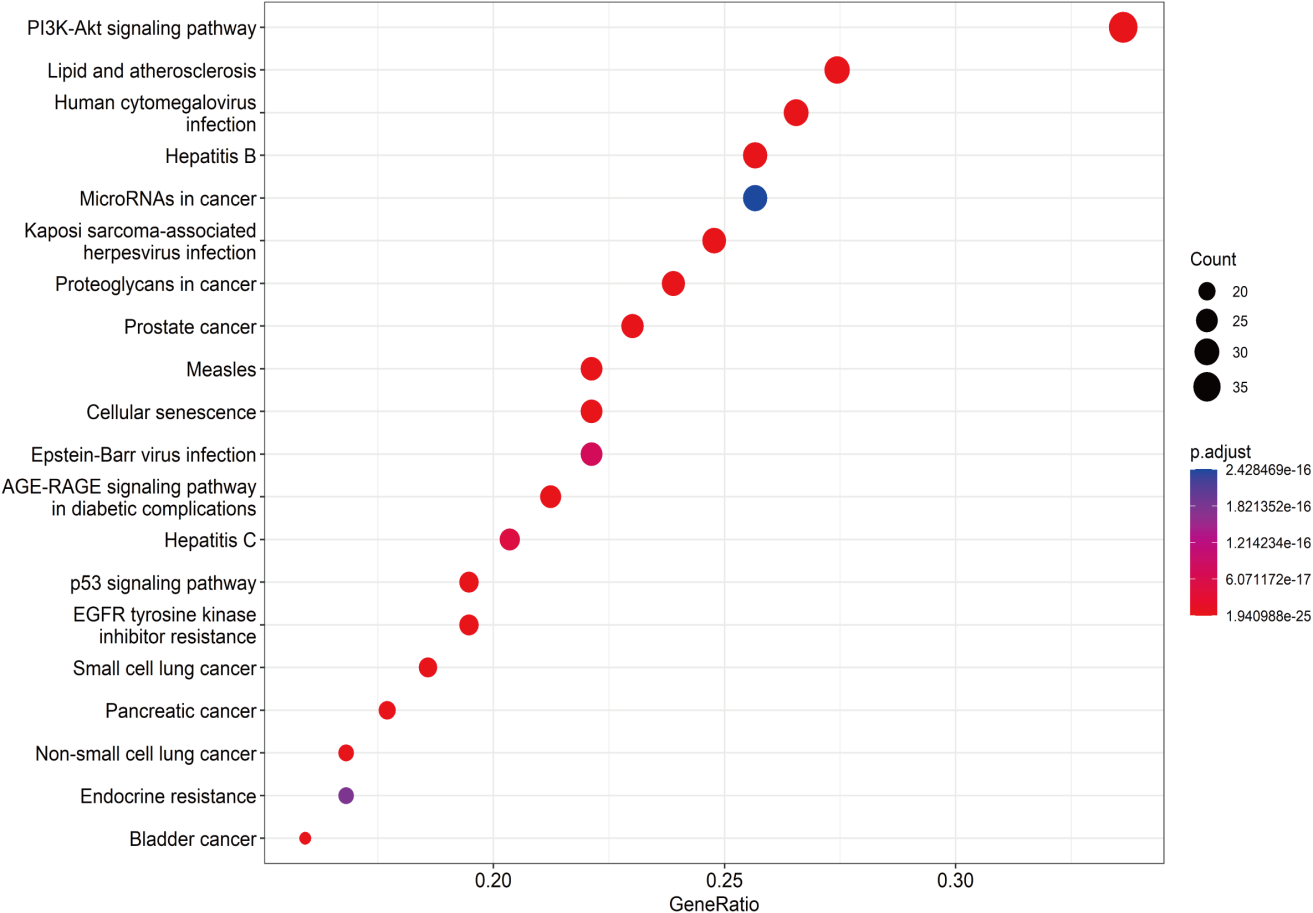
Top 20 KEGG bubble charts.

**Figure 11:**
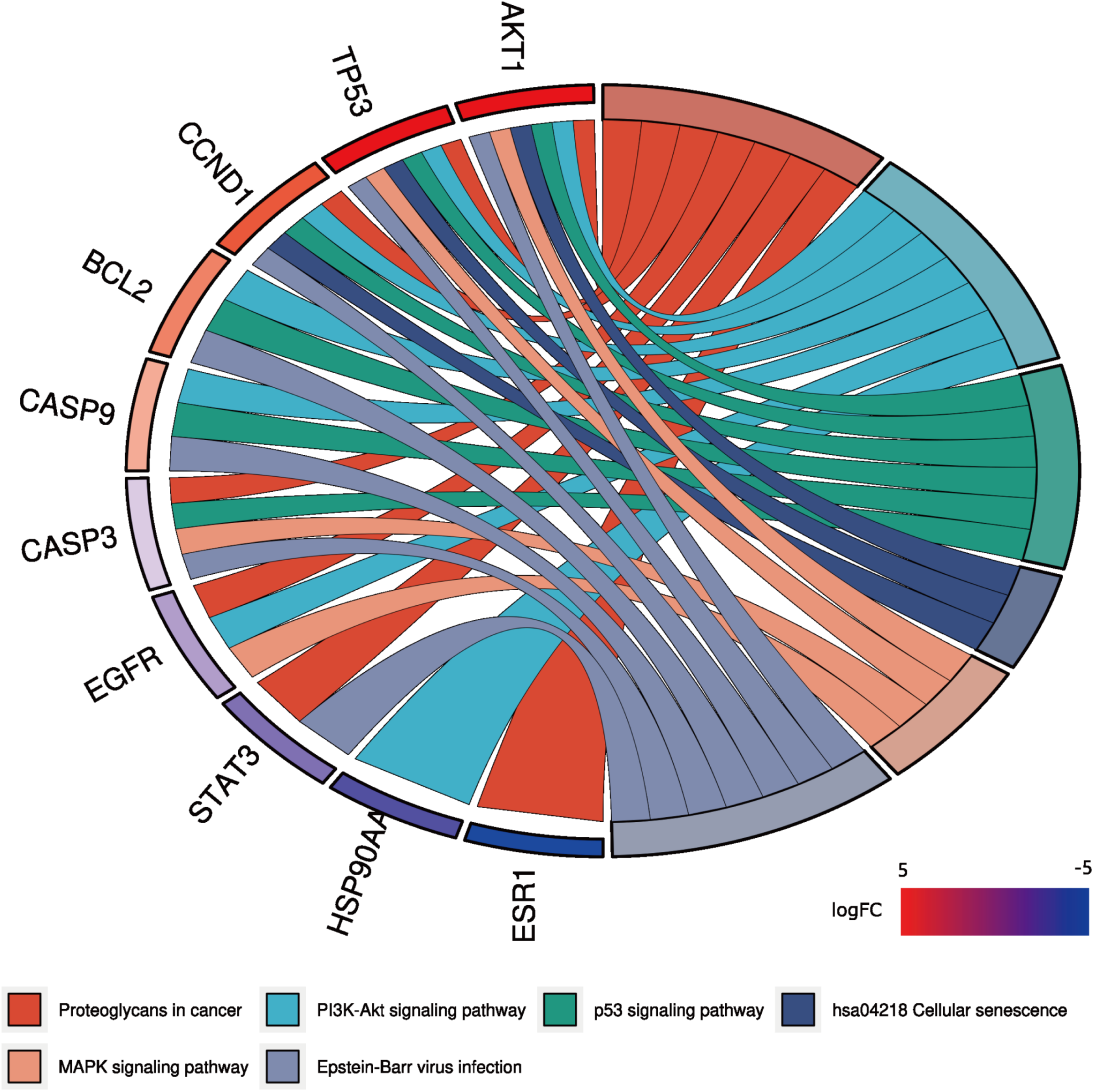
KEGG enrichment analysis chord diagram. Different colors on the right side of the diagram represent different signaling pathways, and the number of bands represents the number of associated genes. One bar on the left represents one gene, and the larger the logFC value, the redder the color.

**Figure 12:**
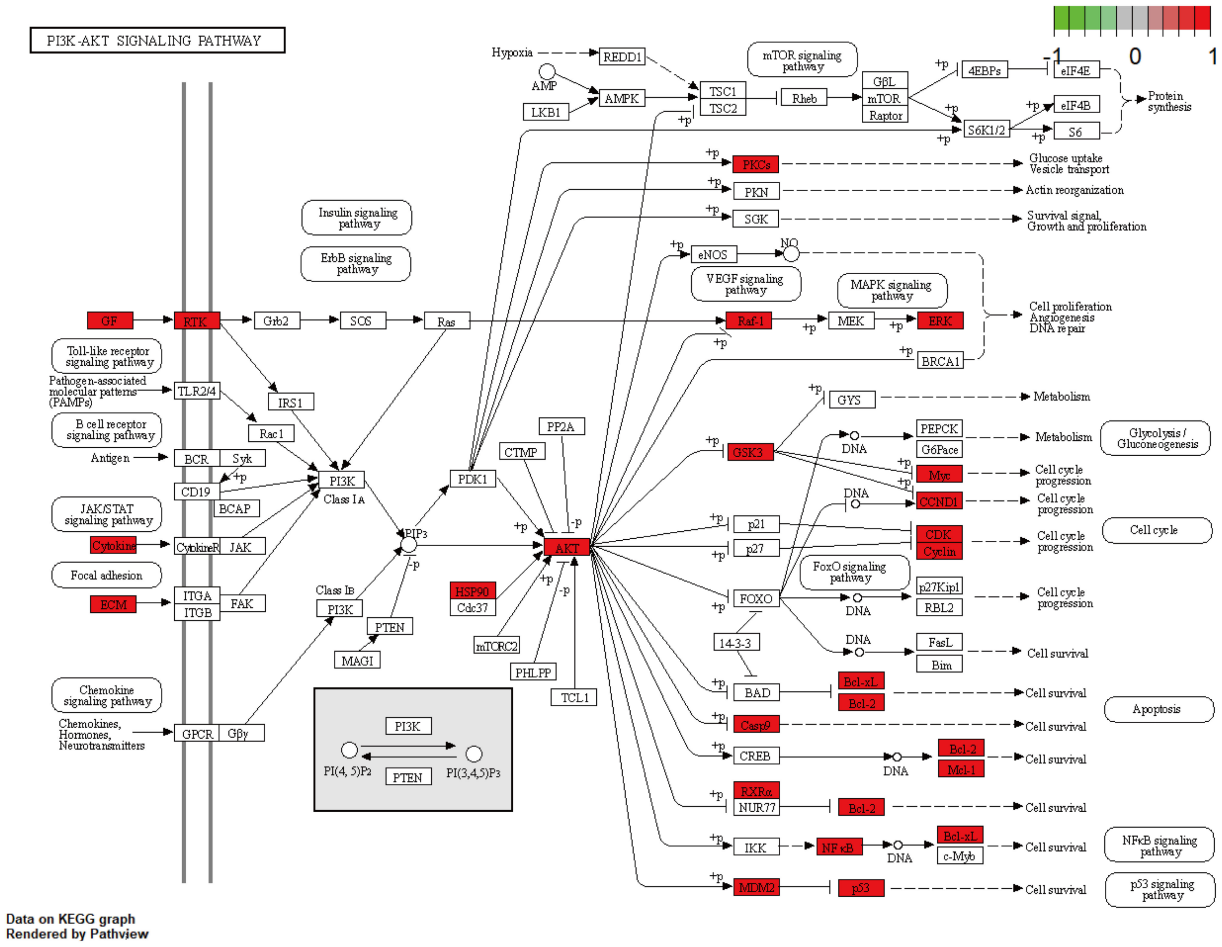
HDH-SBH drug pair act on potential targets and mechanisms of action of PI3K/AKT signaling pathway. Red in the figure represents enriched targets.

A total of 148 KEGG pathways were enriched. Based on the P-values and the number of enriched genes, the top 20 pathways were selected for bar and bubble chart presentations, as shown in **Figures 9**, **10**. These pathways mainly involve the PI3K/AKT signaling pathway, Proteoglycans in cancer, p53 signaling pathway, etc., suggesting that HDH-SBH drug pair may exert anti-NPC effects through multiple cancer-related pathways. Six KEGG pathways related to the development of NPC were chosen for pathway-gene correlation presentation, as seen in **Figure 11**. Key targets like AKT1, TP53, and BCL2 were ranked high, indicating their potential significance in HDH-SBH drug pair’s anti-NPC action. Taking the top-ranked PI3K/AKT signaling pathway from the KEGG enrichment analysis as an example, the potential targets and mechanism of action of HDH-SBH drug pair in treating NPC are depicted in **Figure 12**. Combining results from the PPI graph, KEGG chord diagram, and KEGG core pathways, TP53, AKT1, and BCL2 were selected as core targets for this study.

#### 3.1.6 Molecular docking validation

**Figure 13:**
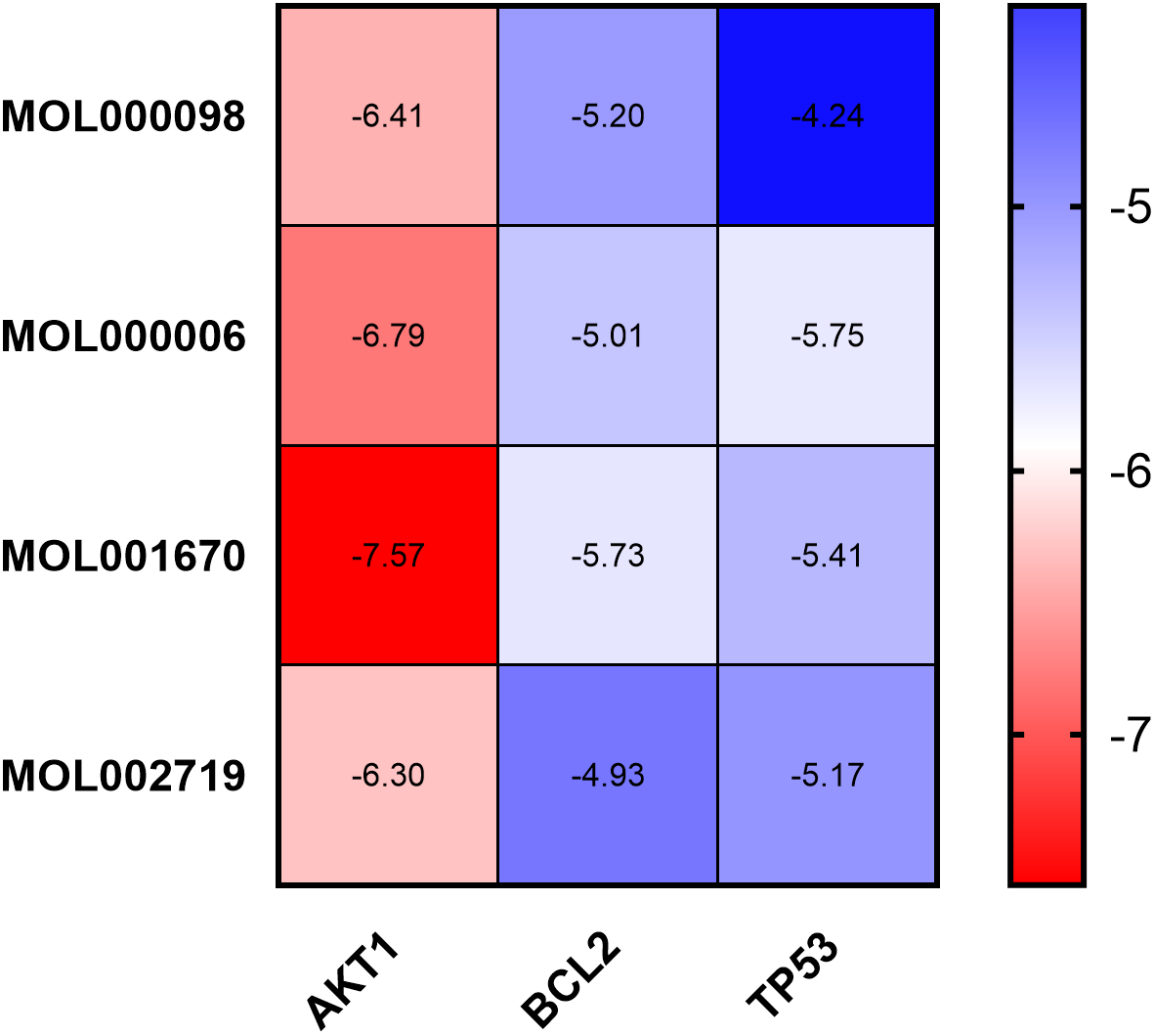
Binding energy thermogram of the four core active ingredients in HDH-SBH drug pair with the three core targets in NPC.

**Figure 14:**
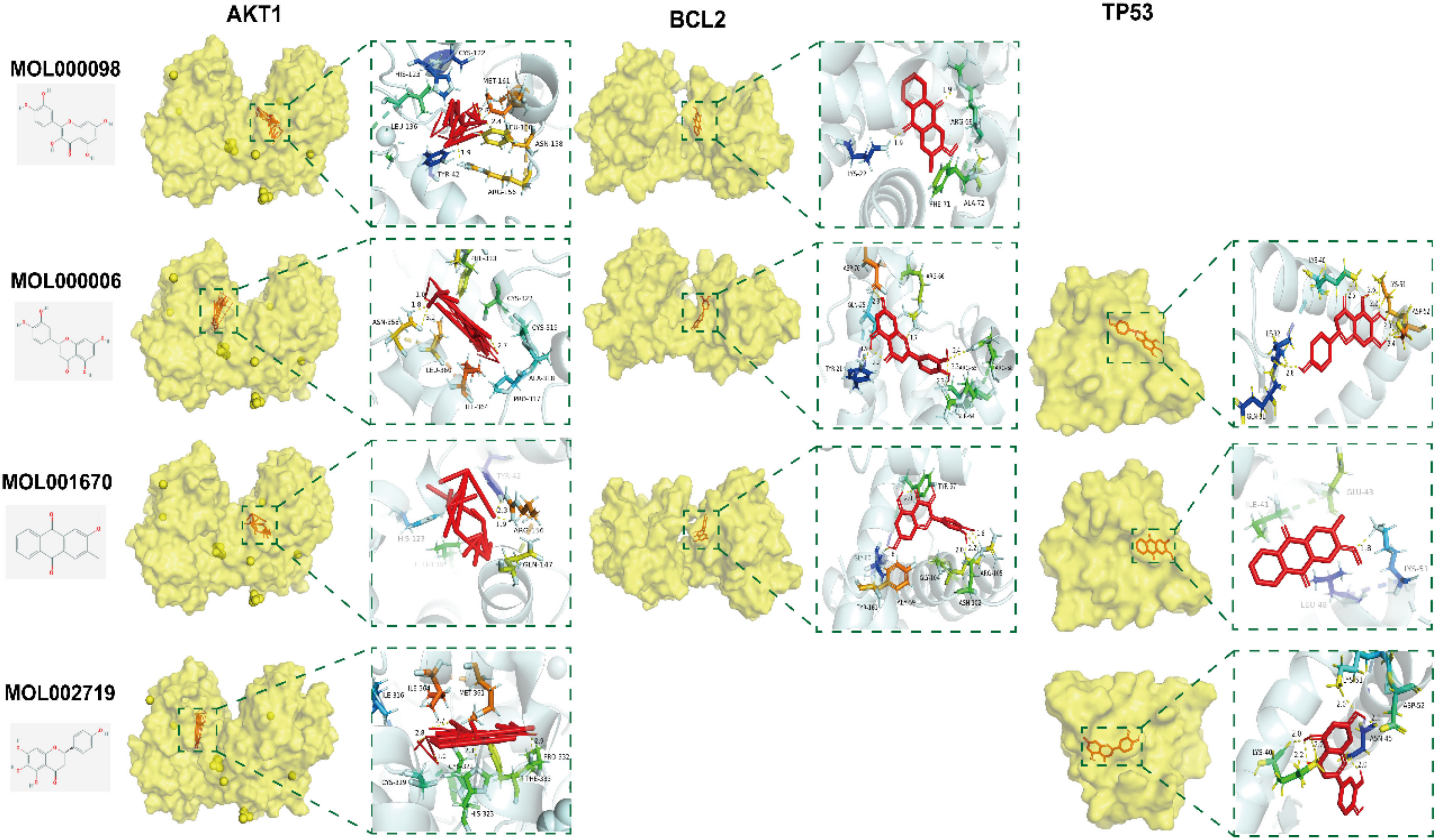
Docking pattern of the four core active ingredients in HDH-SBH drug pair with the three core targets in NPC. Molecular docking and visualization were conducted with AutoDock Tools (version 1.5.6) and PyMOL (version 2.3.1), respectively.

Molecular docking was performed using AutoDock Tools 1.5.6 software on the core active ingredients quercetin, luteolin, 2-methoxy-3-methyl-9,10-anthraquinone, and 6-Hydroxynaringenin, as well as the core targets AKT1, TP53, and BCL2. The docking results were assessed, retaining only the maximum absolute value of affinity. The PDB information of the core target proteins is listed in **Table 3**, and the binding energies are visualized in heatmaps, as shown in **Figure 13**. A binding energy lower than 0 kcal/mol indicates spontaneous binding potential between the protein receptor and the small molecule ligand. Binding energy less than -5 kcal/mol suggests good binding activity between the ligand and receptor protein (20, 21). Lower binding energies and a higher number of hydrogen bonds indicate more stable binding. The docking results revealed that the core active ingredients of HDH-SBH drug pair show good binding activity with the core targets, especially with AKT1, where the binding energy is the lowest, indicating the strongest binding. This suggests that HDH-SBH drug pair might exert anti-NPC effects by acting on these key targets, particularly AKT1. Finally, some of the docking results were visualized using PyMOL 2.3.1 software, as seen in **Figure 14**.

**Table 3:** Information of 3 proteins involved in molecular docking.

Target	Uniprot-ID	PDB-ID	Ligand-ID
AKT1	P31749	1H1O	GOL
BCL2	P10415	2W3L	DRO
TP53	P04637	1A1U	/

### 3.2 Results of *in vitro* experimental validation

#### 3.2.1 Cell viability assay analysis

**Figure 15:**
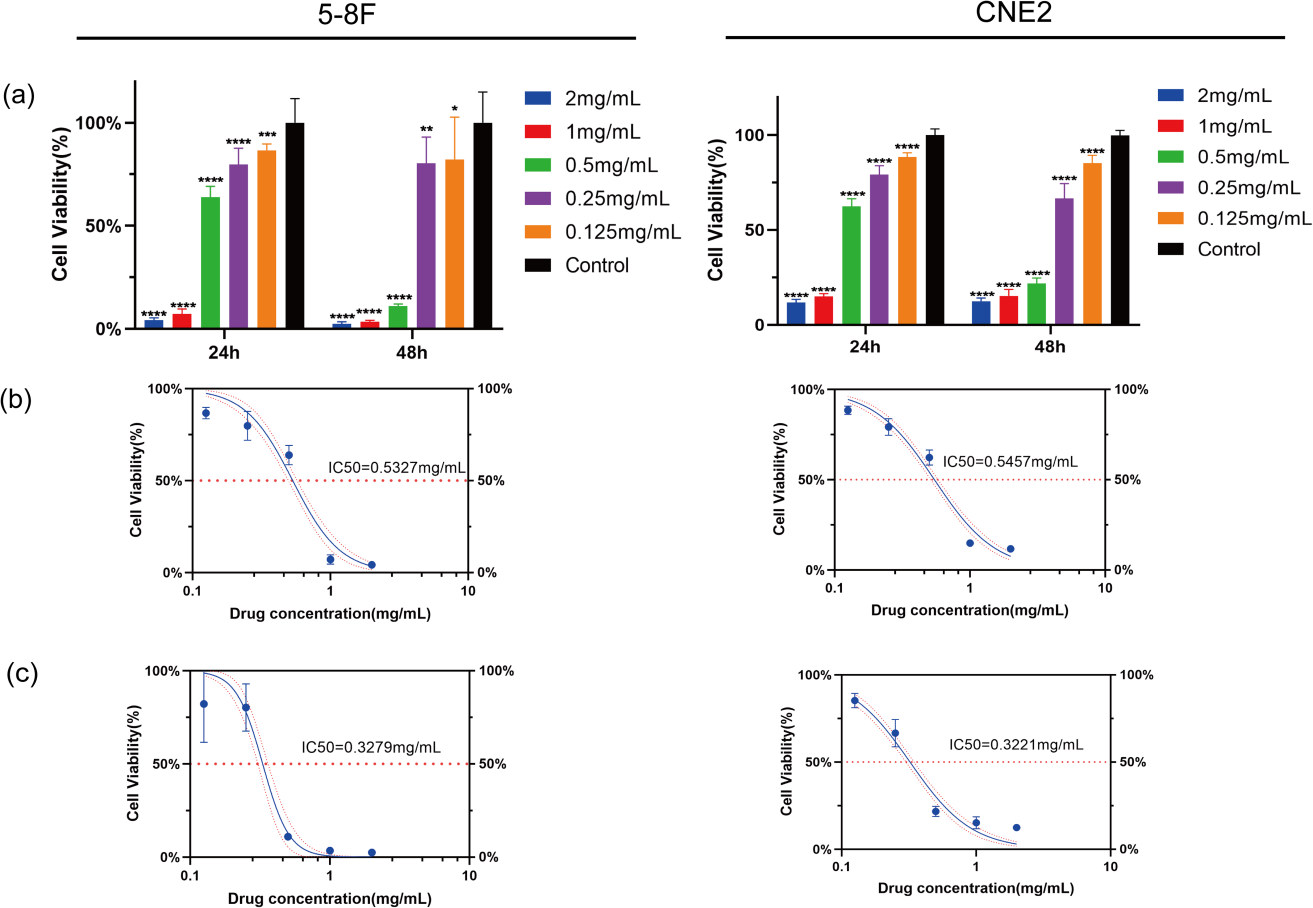
HDH-SBH drug pair inhibited Proliferation in 5-8F cells and CNE2 cells. **(a)** Bar chart of viability rate of 5-8F cells and CNE2 cells treated with different concentrations of HDH-SBH drug pair intervention at two time points (Data are the mean ± SD (n=12). Compared with the Control group, *P<0.05, **P<0.01, ***P<0.001, ****P<0.0001). **(b)** The 24-hour half inhibitory concentration of HDH-SBH drug pair on 5-8F cells and CNE2 cells. **(c)** The 48-hour half inhibitory concentration of HDH-SBH drug pair on 5-8F cells and CNE2 cells. The red horizontal dashed line represents the half inhibitory concentration value, and the red curves represent the 95% confidence interval.

After treatment with HDH-SBH drug pair for 24 and 48 hours, the results indicated a significant inhibitory effect on the proliferation of NPC cells compared to the control group (*P*<0.05), demonstrating concentration dependency. The half-maximal inhibitory concentration (IC_50_) for 5-8F cells at 24 hours and 48 hours was 0.5327 mg/mL and 0.3279 mg/mL, respectively; for CNE2 cells, the IC_50_ at 24 hours and 48 hours was 0.5457 mg/mL and 0.3221 mg/mL, respectively (**Figure 15**). Microscopic observation (40x) after 48 hours revealed that cells in the control group proliferated more rapidly, whereas HDH-SBH drug pair group showed the most significant inhibition of cell vitality. Therefore, the IC_50_ of HDH-SBH drug pair at 48 hours was chosen for intervention in subsequent experiments.

#### 3.2.2 Plate colony formation assay analysis

**Figure 16:**
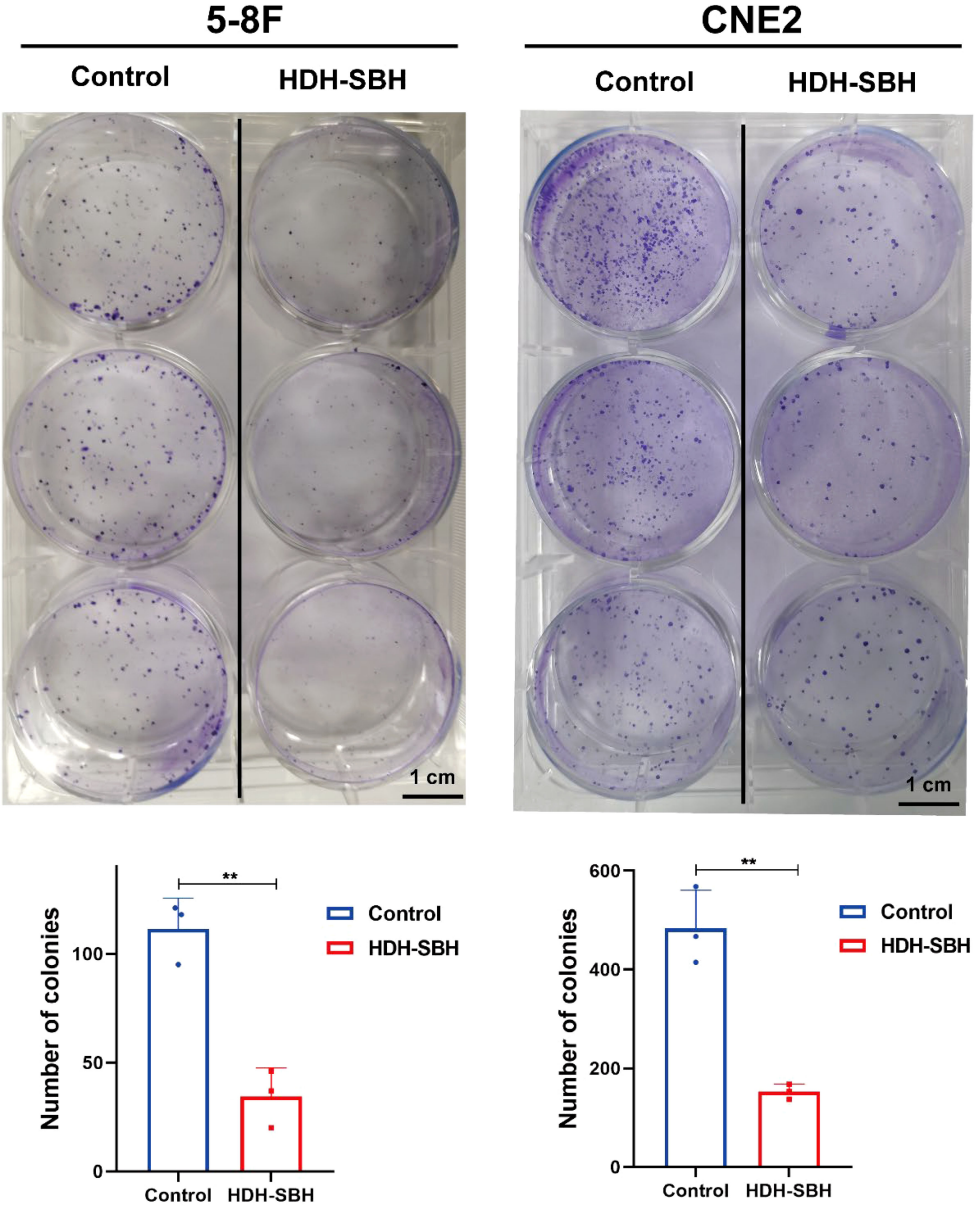
The ability of HDH-SBH drug pair to inhibit colony formation in 5-8F cells and CNE2 cells. (Data are the mean ± SD (n=3). Compared with the Control group, **P<0.01).

The plate colony formation assay was used to assess the effect of HDH-SBH drug pair on the colony-forming ability of 5-8F cells and CNE2 cells. The results showed that after 10 days of culture, the 5-8F cells control group exhibited (111 ± 14.22) cell colonies, while HDH-SBH drug pair treatment group had (32 ± 13.20) cell colonies. For CNE2 cells, the control group had (483.33 ± 77.80) cell colonies, whereas HDH-SBH treatment group displayed (152.67 ± 15.50) cell colonies. The difference between the two groups was statistically significant (*P*<0.01), indicating that HDH-SBH drug pair significantly reduced the colony formation ability of NPC cells (**Figure 16**).

#### 3.2.3 Migration ability analysis

**Figure 17:**
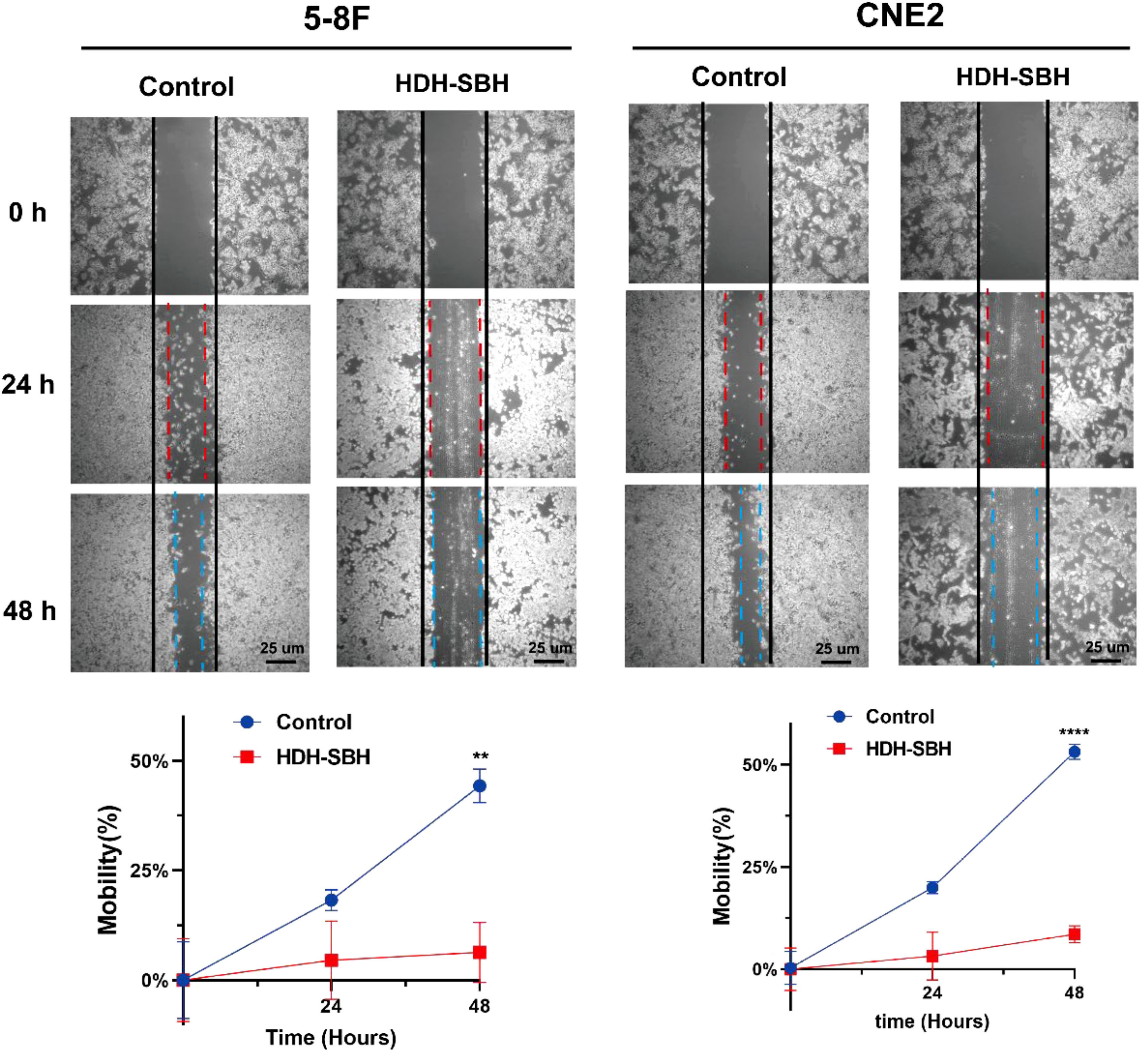
The effect of HDH-SBH drug pair on 5-8F cells and CNE2 cells in wounding experiments. (Data are the mean ± SD (n=3). Compared with the Control group, **P<0.01, ****P<0.0001).

The wound healing assay was performed to evaluate the migration ability of 5-8F cells and CNE2 after treatment with HDH-SBH drug pair. The results demonstrated that in 5-8F cells, the migration rate of the control group was (18.26 ± 2.41)% at 24 hours and (44.29 ± 3.77)% at 48 hours. For HDH-SBH drug pair treatment group, the migration rate was (4.55 ± 8.92)% at 24 hours and (6.36 ± 6.83)% at 48 hours. In CNE2 cells, the control group’s migration rate was (19.96 ± 1.50)% at 24 hours and (53.16 ± 1.84)% at 48 hours, while for HDH-SBH drug pair treatment group, it was (3.22 ± 5.86)% at 24 hours and (8.55 ± 2.06)% at 48 hours. A significant difference was observed at 48 hours in both NPC cell lines (*P*<0.01), indicating that HDH-SBH drug pair treatment progressively reduced the migration capacity of NPC cells over time (**Figure 17**).

#### 3.2.4 Analysis of core target mRNA expression

**Figure 18:**
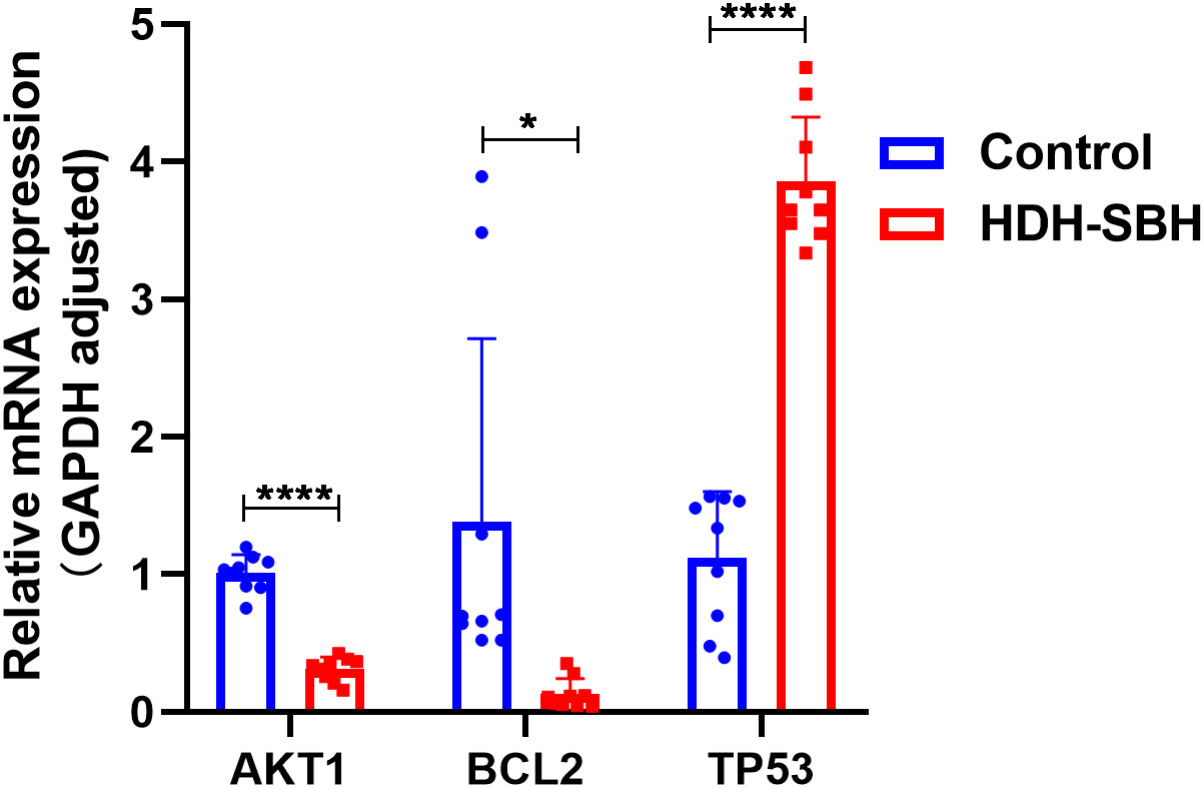
The effect of HDH-SBH drug pair on mRNA expression of core targets in 5-8F cells. (Data are the mean ± SD (n=3). Compared with the Control group, *P<0.05, ****P<0.0001).

To validate the enriched core signaling pathway, PI3K/AKT, qRT-PCR was conducted to analyze the mRNA expression of core targets. The results showed that, compared with the control group, the expression of AKT1 and BCL2 mRNA in HDH-SBH drug pair group (0.3279 mg/mL) significantly decreased, while the expression of TP53 significantly increased, with the differences being statistically significant (*P*<0.05) (**Figure 18**).

#### 3.2.5 Analysis of core target protein expression

**Figure 19:**
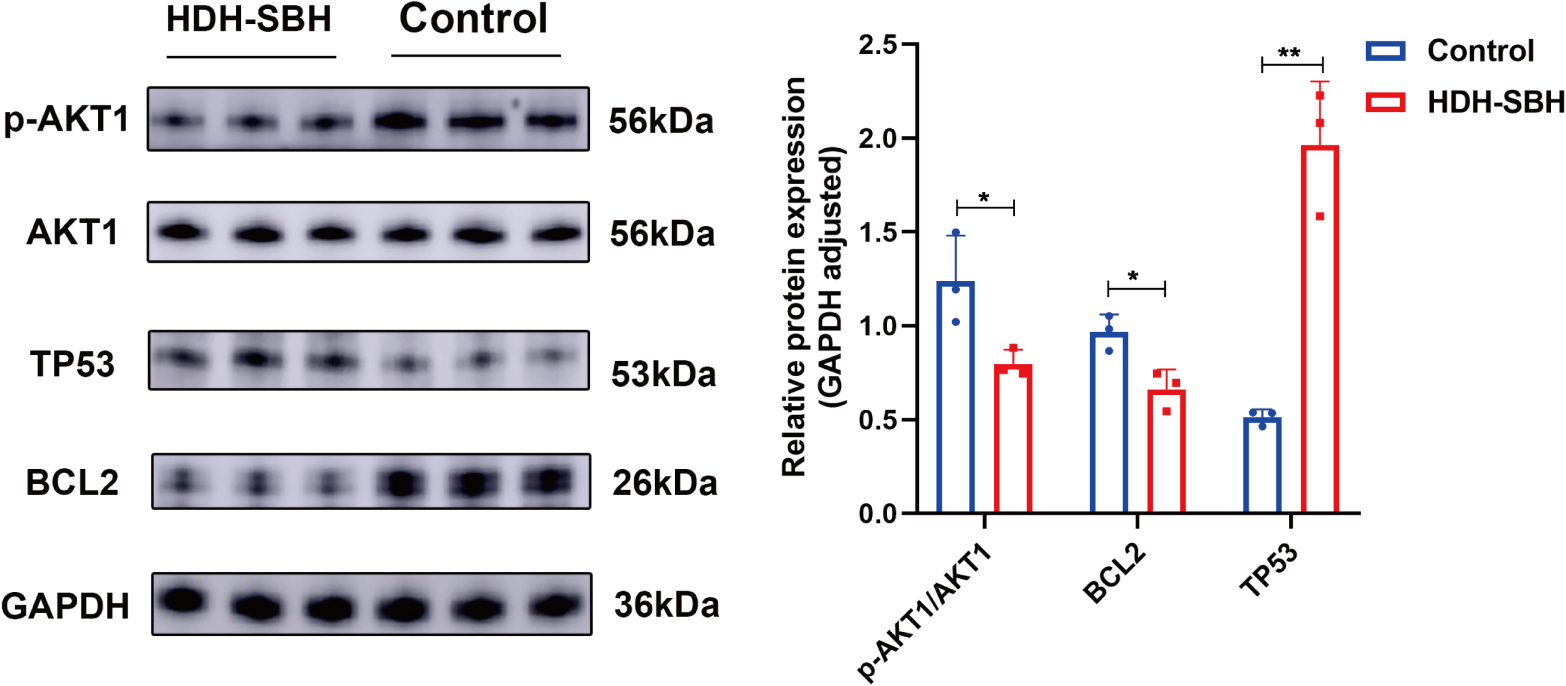
The effect of HDH-SBH drug pair on protein expression of core targets in 5-8F cells. (Data are the mean ± SD (n=3). Compared with the Control group, *P<0.05, **P<0.01).

To validate the enrichment of the core signaling pathway, PI3K/AKT, a Western blot assay was conducted to assess the protein expression of core targets. Given that AKT must be phosphorylated to be functional, the expression of both AKT1 and its phosphorylated form, p-AKT1, was examined. The ratio of AKT1/p-AKT1 was calculated and presented as the outcome. The results indicated a significant decrease in the protein expression of AKT1 and BCL2 in HDH-SBH drug pair group (0.3279 mg/mL) compared to the control group, whereas the expression of TP53 significantly increased, with these differences being statistically significant (*P*<0.05). This suggests that HDH-SBH drug pair may exert its anti-NPC effects by inhibiting the expression of the PI3K/AKT signaling pathway (**Figure 19**).

#### 3.2.6 Cell apoptosis analysis

**Figure 20:**
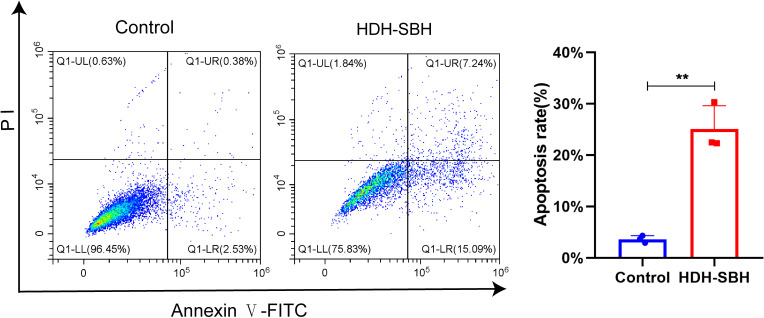
The effect of HDH-SBH drug pair on 5-8F cells in apoptosis. (Data are the mean ± SD (n=3). Compared with the Control group, **P<0.01).

Flow cytometry was used to examine the apoptosis of 5-8F cells following treatment with HDH-SBH drug pair. The results showed that the apoptosis rates for HDH-SBH drug pair group and the control group were (3.62 ± 0.70) % and (25.04 ± 4.58) %, respectively, with HDH-SBH drug pair group exhibiting a higher rate of apoptosis than the control group (*P*<0.05) (**Figure 20**). Observation under the microscope (400x) of the morphological changes associated with apoptosis revealed that cells treated with HDH-SBH drug pair were fewer in number, smaller in size, showed morphological shrinkage, and increased cell rupture. This suggests that one of the mechanisms by which HDH-SBH drug pair antagonizes NPC may involve mediating tumor cell apoptosis.

## 4 Discussion

The aggressive nature of undifferentiated NPC, characterized by pronounced malignancy and metastatic propensity, underscores the urgent need for molecular insights to complement radiation therapy’s central role (22, 23). Hence, it becomes imperative to unravel the molecular underpinnings that are intrinsically linked to the tumor’s prognostic landscape and invasive demeanor. The advent and subsequent proliferation of high-throughput sequencing alongside microarray modalities have significantly propelled the exploration of differential gene expression landscapes intimately associated with NPC, thereby paving novel avenues for the identification of viable diagnostic and therapeutic targets aimed at the disease’s mitigation and management (24, 25). Our integrated approach combining GEO database analysis (240 differential genes) with network pharmacology revealed HDH-SBH’s 36 active ingredients and 115 shared targets. Through 2182 GO terms and 148 KEGG pathways, the PI3K/AKT pathway emerged as the central mechanism, with molecular docking demonstrating strong interactions between quercetin, luteolin, 2-methoxy-3-methyl-9,10-anthraquinone, 6-Hydroxynaringenin, and core targets TP53/AKT1/BCL2. Complementary *in vitro* assays validated these findings, establishing a multi-modal methodology bridging bioinformatics and experimental validation.

The “Drug-Ingredient-Target-Disease” network identified four key components. Quercetin, a multifunctional flavonoid, synergizes with cisplatin (combination index <1) in NPC through VEGF and NF-κB suppression, while inducing autophagy and senescence to overcome drug resistance (26–29). Luteolin exerts dual anti-NPC effects: G1-phase cell cycle arrest via Akt/GSK-3β/cyclin D1 axis (37) and EBV lytic gene suppression (30). 2-methoxy-3-methyl-9,10-anthraquinone enhances glucose metabolism through AMPK activation (31), while 6-Hydroxynaringenin (scutellarein) promotes apoptosis via CDC4-mediated RAGE ubiquitination (32). This combinatorial strategy targets multiple resistance pathways while maintaining traditional pharmacological wisdom.

Network pharmacology identified AKT1, BCL2, and TP53 as pivotal targets. AKT1, the central PI3K-AKT-mTOR pathway node, drives NPC metastasis and therapy resistance (33, 34). Its phosphorylation at Ser473 facilitates epithelial-mesenchymal transition, while EBV-LMP1 enhances cyclophilin A/AKT1 interactions to promote tumor progression (35, 36). The role of BCL2, a pro-survival protein encoded by the bcl-2 proto-oncogene, is instrumental in inhibiting apoptosis by preventing cytochrome c release from mitochondria (37, 38). Research initiatives, such as those by Xiao et al., have demonstrated Ginkgolic Acid’s capacity to dose-dependently suppress BCL2 expression, thereby promoting apoptosis in NPC cells, with synergistic effects observed when combined with 5-fluorouracil (39). TP53, encoded by the tumor suppressor gene TP53 on chromosome 17 and known for its molecular weight of 53 kDa, is arguably the most frequently mutated gene across human cancers (40). The profound implications of TP53 mutations on tumor biology, coupled with its tumor-suppressive function, spotlight it as a highly attractive target for cancer therapy (41–43). Meta-analytical evidence by Yang et al., encompassing studies of 1189 patients, underscores a significant association between positive TP53 status and diminished 5-year survival rates in NPC patients, thereby affirming TP53’s relevance in NPC prognosis and treatment strategies (44).

Functional enrichment revealed HDH-SBH’s modulation of oxidative stress response and kinase activities. KEGG analysis confirmed PI3K/AKT pathway dominance, where PI3K-generated PIP3 recruits AKT to regulate survival and metastasis (45, 46). Extensive research has demonstrated a close association between the PI3K/AKT signaling pathway and the pathogenesis, progression, and prognosis of NPC (47–49). Our *in vitro* data demonstrated HDH-SBH’s tripartite effects: 1) inhibition of proliferation and migration, 2) induction of apoptosis (with reduced BCL2 expression), and 3) PI3K/AKT pathway modulation (elevated AKT phosphorylation and increased TP53 expression; *P*<0.05). This suite of experiments resonates with previous studies, affirming that both HDH, SBH, and their constituent active ingredients can curtail cell growth and migration across various cancers, including lung, colorectal, and liver cancers, and precipitate apoptosis (14, 50, 51). Collectively, these findings herald HDH-SBH drug pair as a formidable anti-tumor agent against NPC, primarily by modulating the PI3K/AKT signaling pathway. The strategic reduction in AKT1 and BCL2 expression or phosphorylation, coupled with the amplification of TP53 expression and the consequent inhibition of pathway activation, delineates a promising therapeutic avenue against NPC, reinforcing the drug pair’s potential in cancer therapy.

Our *in vitro* experiments demonstrated significant inhibitory effects of the HDH-SBH combination on 5-8F and CNE2 NPC cell lines. However, emerging evidence raises critical concerns about cell line authenticity in NPC research. Xu et al. recently reported that 1,159 studies published between 2000 and 2023 employed misidentified NPC cell lines contaminated with HeLa cells (52), underscoring the imperative for rigorous authentication in preclinical models. To address this systemic issue, we conducted short tandem repeat (STR) profiling prior to experimentation, which confirmed the genetic integrity of both 5-8F and CNE2 cell lines without evidence of HeLa cross-contamination. These measures ensure the biological relevance of our findings while highlighting the necessity for standardized authentication protocols in NPC research.

Despite these methodological safeguards, several limitations warrant consideration. First, while network pharmacology provides a systematic framework for target prediction, its accuracy remains contingent on database completeness and algorithm biases. Rapidly evolving annotations of gene functions and interactions may introduce temporal limitations to our computational models. Second, although STR-authenticated 5-8F and CNE2 cell lines were rigorously employed, their use in monoculture systems inherently restricts recapitulation of NPC tumor heterogeneity and microenvironmental dynamics observed in clinical specimens. Third, the absence of longitudinal experimental data precludes assessment of HDH-SBH’s chronic effects. This is particularly relevant given clinical reports of long-term adverse reactions to similar herbal formulations, including gastrointestinal toxicity, hepatorenal impairment, and hemorrhagic risks (**Table 4**).

**Table 4:** List of abbreviations.

Abbreviation	Abbreviation Full Form
ADME	absorption, distribution, metabolism, and excretion
BC	betweenness centrality
BP	biological process
CC	cellular components
CCK-8	Cell Counting Kit-8
CI	combination index
DC	degree centrality
DL	drug-likeness
EMT	epithelial-mesenchymal transition
FBS	Fetal bovine serum
GEO	Gene Expression Omnibus
GO	Gene Ontology
HDH	*Hedyotis Diffusae Herba*
KEGG	Kyoto Encyclopedia of Genes and Genomes
MF	molecular function
NPC	Nasopharyngeal carcinoma
OB	oral bioavailability
OMIM	Online Mendelian Inheritance in Man
PPI	Protein-Protein Interaction
SBH	*Scutellariae Barbatae Herba*
TCM	Traditional Chinese Medicine
TCMSP	Traditional Chinese Medicine Systems Pharmacology Database and Analysis Platform

## 5 Conclusion

This investigation, through the synergistic application of GEO database exploration and network pharmacology methodologies, illuminated the conjectural molecular underpinnings through which HDH-SBH drug pair exerts therapeutic effects against NPC. Validation of these mechanistic insights was achieved via *in vitro* cellular assays. The results compellingly suggest that HDH-SBH drug pair suppresses cellular proliferation and migration while promoting apoptosis in 5-8F cells and CNE2 cells, activities closely intertwined with the dysregulated modulation of pivotal targets within the PI3K/AKT signaling pathway, notably AKT1, TP53, and BCL2. The research posits that the mechanism underlying HDH-SBH drug pair’s anticancer efficacy against NPC may pivot on the suppression of PI3K/AKT signaling pathway activation. This elucidation not only advances our comprehension of the molecular dynamics driving HDH-SBH drug pair’s therapeutic intervention in NPC but also pioneers an efficacious paradigm for the development and clinical deployment of anticancer therapeutics derived from the critical active constituents of HDH-SBH drug pair. Through this multifaceted approach, the study contributes significantly to the burgeoning field of targeted cancer therapy, offering a promising avenue for novel drug discovery and tailored treatment strategies against NPC, thereby underscoring the potential of integrating traditional herbal medicine with contemporary oncological research. While these findings underscore the therapeutic potential of HDH-SBH drug pair in NPC, future studies must prioritize translating these *in vitro* results into clinically actionable strategies. Animal models mimicking NPC progression and metastasis are essential to validate the efficacy and safety of HDH-SBH drug pair *in vivo*. Pharmacokinetic studies should be conducted to optimize dosing regimens and evaluate systemic bioavailability. Furthermore, clinical trials are imperative to assess the feasibility of integrating HDH-SBH drug pair with existing chemoradiotherapy regimens, particularly to determine its synergistic effects and ability to mitigate treatment-related toxicities. The development of standardized HDH-SBH formulations and the identification of biomarkers predictive of therapeutic response will be critical steps toward personalized NPC therapy. Only through such translational efforts can the promise of HDH-SBH drug pair transition from bench to bedside, ultimately improving outcomes for NPC patients.

## Data Availability

The original contributions presented in the study are included in the article/supplementary material. Further inquiries can be directed to the corresponding authors.
